# A Biomarker‐Based Classification of Corticobasal Syndrome

**DOI:** 10.1002/mds.70070

**Published:** 2025-10-06

**Authors:** Carla Palleis, Alexander Maximilian Bernhardt, Endy Weidinger, Urban M. Fietzek, Alexander Jäck, Sabrina Katzdobler, Johannes Gnörich, Theresa Bauer, Nicolai Franzmeier, Robert Perneczky, German Imaging Initiative for Tauopathies (GII4T), Matthias Brendel, Johannes Levin, Günter U. Höglinger

**Affiliations:** ^1^ Department of Neurology LMU University Hospital, Ludwig‐Maximilians‐Universität (LMU) München Munich Germany; ^2^ Munich Cluster for Systems Neurology (SyNergy) Munich Germany; ^3^ German Center for Neurodegenerative Diseases (DZNE) Munich Munich Germany; ^4^ Schön Klinik München Schwabing Munich Germany; ^5^ Department of Nuclear Medicine LMU University Hospital, LMU Munich Munich Germany; ^6^ Institute for Stroke and Dementia Research (ISD), LMU University Hospital, Ludwig‐Maximilians‐Universität (LMU) München Munich Germany; ^7^ University of Gothenburg, The Sahlgrenska Academy, Institute of Neuroscience and Physiology Department of Psychiatry and Neurochemistry Gothenburg Sweden; ^8^ Department of Psychiatry and Psychotherapy, University Hospital Ludwig‐Maximilians‐Universität Munich Germany; ^9^ Sheffield Institute for Translational Neuroscience (SITrN), University of Sheffield Sheffield UK; ^10^ Ageing Epidemiology Research Unit (AGE), School of Public Health, Imperial College London UK

**Keywords:** proteinopathies, α‐synuclein seed amplification assay, tau‐PET, β‐amyloid

## Abstract

**Background:**

Corticobasal syndrome (CBS) is a clinically defined syndrome with progressive movement and cortical dysfunction, caused by various underlying pathologies, most commonly tau‐predominant pathologies such as progressive supranuclear palsy and corticobasal degeneration, or Alzheimer's disease (AD). Lewy‐type α‐synucleinopathies (LTS), TDP‐43 proteinopathies, and mixed pathologies may also underlie CBS. The clinical impact of these pathologies remains poorly understood.

**Objectives:**

To subclassify CBS patients in vivo using biomarkers for amyloid‐β (Aβ), Tau, and α‐synuclein (αSyn), and assess the clinical relevance of this stratification.

**Methods:**

We conducted a prospective cohort study of 50 CBS patients at LMU University Hospital Munich. Biomarker analysis included cerebrospinal fluid (CSF) Aβ42 and Aβ42/40, [^18^F]flutemetamol Aβ‐PET, [^18^F]PI‐2620 tau‐PET, and αSyn seed amplification assays in CSF. CSF neurofilament light chain (NfL) served as a marker of neurodegeneration. Patients were stratified into six groups based on biomarker positivity.

**Results:**

Tau positivity was found in 90% of CBS cases, Aβ in 28%, and αSyn in 24%. Stratification identified: 52% consistent with tau‐predominant pathology, 18% with AD, 10% with AD+LTS, 10% with tau‐predominant+LTS, 4% with isolated LTS, and 6% unclassified. αSyn positivity was more frequent in AD‐CBS (36%) than in tau‐predominant‐CBS (16%). Aβ‐positive cases showed greater cognitive impairment; Tau positivity correlated with worse motor symptoms; αSyn‐positive patients had milder motor symptoms, slower progression, and lower NfL levels.

**Conclusions:**

CBS is molecularly heterogeneous. Biomarker‐based classification may enhance diagnostic precision and support personalized therapeutic strategies. © 2025 The Author(s). *Movement Disorders* published by Wiley Periodicals LLC on behalf of International Parkinson and Movement Disorder Society.

Corticobasal syndrome (CBS) is a clinically defined condition characterized by progressive cortical and basal ganglia dysfunction, manifesting as cognitive and motor impairments.^1^, ^2^, ^3^ While CBS presents as a clinically defined syndrome, it can result from diverse neuropathologies.^4^, ^5^, ^6^ Most commonly, CBS is linked to tau‐predominant pathologies, often involving 4‐repeat tau (4RT) such as progressive supranuclear palsy (PSP) and corticobasal degeneration (CBD) (~50%), followed by Alzheimer's disease (AD) with β‐amyloid (Aβ) pathology and 3‐repeat/4‐repeat (3R/4R) tau pathology (~25–40%). Less frequently, Lewy‐type α‐synucleinopathy (LTS), TDP‐43 proteinopathies, or mixed pathologies contribute (~12–30%).^4^, ^6^, ^7^ CBD refers to a distinct histopathological entity and should not be equated with CBS.

The clinical relevance of underlying pathologies in CBS remains poorly understood due to its rarity and reliance on retrospective autopsy series. As disease‐modifying treatments emerge,^3^, ^8^ molecular stratification during life is critical to enable personalized, pathology‐specific therapies and prospective biomarker‐based studies are urgently needed. Other neurodegenerative diseases have adopted biological definitions. In AD, the ATN classification system^9^ and its expansion (ATNIVS)^10^ incorporate biomarkers for Aβ, tau, neurodegeneration, inflammation, vascular disease, and α‐synuclein. Parkinson's disease frameworks such as SynNeurGe^11^ and NSD‐ISS^12^ also include α‐synuclein, neurodegeneration, and genetics. In these frameworks, cerebrospinal fluid (CSF) α‐synuclein seed amplification assays (SAA) are increasingly central. In CBS, molecular stratification is now feasible using biomarkers: Aβ pathology can be detected via CSF Aβ42/40 or amyloid‐PET^9^, ^10^; tau pathology by [^18^F]PI‐2620 PET^13^, ^14^, ^15^, ^16^, ^17^, ^18^, ^19^, ^20^; and LTS by CSF αSyn SAA.^21^, ^22^, ^23^, ^24^

Here, we report the first prospective biomarker‐based subclassification of CBS. We assessed the prevalence and co‐occurrence of Aβ, tau, and α‐synuclein pathologies, and examined their associations with clinical severity and progression. Hypothesis‐driven analyses were restricted to five prespecified outcomes (PSP rating scale {PSPRS], Montreal Cognitive Assessment [MoCA], Dementia Apraxia Test [DATE], CSF neurofilament light chain [NfL], CSF Aβ42/40), while all other evaluations were considered exploratory.

## Methods

### Participants and Clinical Assessments

All study‐related procedures were approved by the Ludwig‐Maximilians‐Universität (LMU) Munich ethics committee (ethics applications: 23‐0602, 17‐569, and 19‐022) and the German radiation protection authorities (BfS application: Z5‐22464/2017‐047‐K‐G). All patients provided written informed consent for all study‐related procedures including clinical assessment, lumbar puncture, and positron emission tomography (PET) imaging in accordance with the Declaration of Helsinki and its amendments.

Patients were recruited and prospectively phenotyped at the Department of Neurology at LMU Hospital Munich between February 2018 and March 2024. They were diagnosed by movement disorders specialists as CBS phenotype as defined by the International Parkinson and Movement Disorder Society (MDS) criteria (suggestive of or possible PSP‐CBS phenotype)^1^ and the Armstrong criteria (possible or probable CBD‐CBS).^2^ Other predominant PSP or CBD phenotypes were not included in this study. Clinical assessments included the PSPRS^25^ for characteristic features of 4R‐tauopathies, the MoCA scale^26^ for cognitive impairments, and the DATE^27^ for buccofacial and limb apraxia. Disease duration was defined as time between symptom onset and study visit. Details of the study cohort, assessment of NfL, and genetic testing are provided in the Supplementary Materials.

### Biomarker Assessments

#### Aβ Status

Aβ positivity was determined by an Aβ_42/40_ ratio < 5.5% in CSF^28^ and/or [^18^F]flutemetamol‐PET visual read, blinded to clinical information, was performed as described previously.^29^, ^30^ The amyloid status of each patient was hence either defined as being positive (Aβ^+^) or negative (Aβ^−^). In three patients, Aβ_42/40_ ratio was <5.5%, but [^18^F]flutemetamol‐PET remained negative. These three patients were still classified as Aβ^+^ due to abnormal Core 1 biomarker for AD criteria,^10^ suggesting “CSF‐first” versus “PET‐first” Aβ abnormality.^31^, ^32^ All patients with positive [^18^F]flutemetamol‐PET had an abnormal Aβ_42/40_ ratio.

#### Tau Status

[^18^F]PI‐2620 tau‐PET procedures were performed as described previously.^13^, ^14^ Briefly, patients were scanned at the Department of Nuclear Medicine, LMU Hospital Munich, using a Biograph 64 PET/CT scanner or a harmonized Biograph mCT (Siemens, Erlangen, Germany). Dynamic [^18^F]PI‐2620‐PET (average dose: 188 ± 15 MBq) with emission recording obtained 0–60 min after injection. Static frames of the late phase (20–40 min) were reconstructed for assessing Tau binding.^33^ A dichotomous visual read of prescaled maps of the cortex, basal ganglia, midbrain, and dentate nuclei (ie, standardized uptake value ratio images; range 1.0–2.0; inferior cerebellar reference region) was performed by expert readers blinded to clinical and biomarker information.^13^, ^14^, ^17^ The Tau status of each patient was either defined as being positive (Tau^+^) or negative (Tau^−^).

#### αSyn Status

Lewy‐fold‐specific CSF αSyn SAA was performed as described previously.^24^ A patient sample was classified as positive if at least two of four replicates showed a positive signal. Samples with no signal were defined as negative. Samples with one positive out of four replicates were considered inconclusive and repeated once. If the result remained one of four, the sample was declared inconclusive. For all patients, two samples were tested blinded to clinical information. In our cohort, no samples remained inconclusive and were either defined as positive (αSyn^+^) or negative (αSyn^−^).

### Classification by Disease Entities Based on Biomarker‐Profiles

**FIGURE 1:**
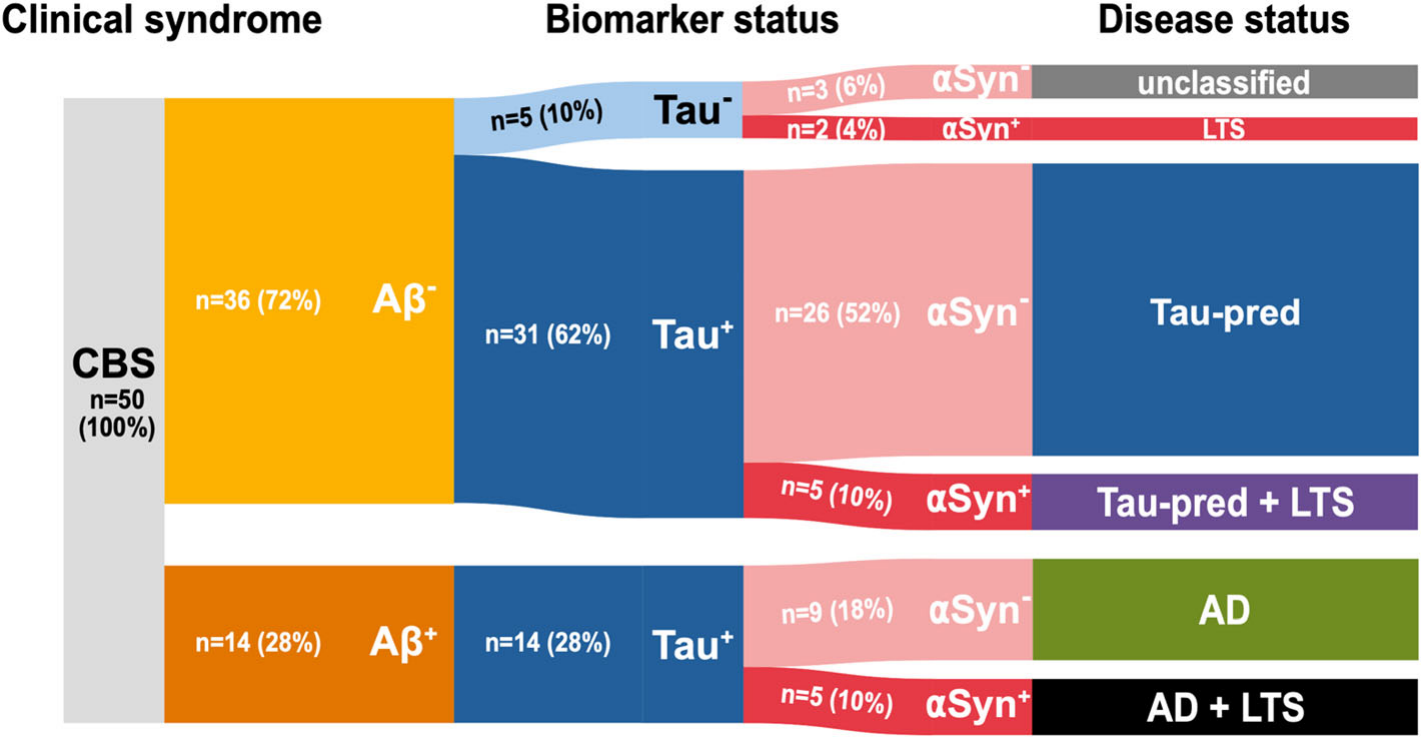
A biomarker‐based classification of corticobasal syndromes (CBS). Aβ^+^, amyloid‐β‐positive; Aβ^−^, amyloid‐β‐negative; αSyn^+^, α‐synuclein‐positive; αSyn^−^, α‐synuclein‐negative; Tau^+^, tau‐positive; Tau^−^, tau‐negative; Tau‐pred, tau‐predominant pathology; AD, Alzheimer's disease; LTS, Lewy‐type synucleinopathy. Patients were screened for Aβ by amyloid‐PET (positron emission tomography) or cerebrospinal fluid (CSF) and denoted as Aβ^−^ in light yellow or Aβ^+^ in dark yellow if one of these measurements showed pathological results. Tau‐PET was employed to stratify patients into Tau^−^ in light blue and Tau^+^ in dark blue. αSyn seed amplification assay from CSF was employed to categorize patients into αSyn^−^ and αSyn^+^ displayed in light red and dark red, respectively. The screening of Aβ, Tau, and αSyn in our 50 CBS patients led to six groups with different presumed underlying pathologies, depending on the observed combination of Aβ, Tau, and αSyn, respectively. The group sizes in the Sankey chart are based on their percentage within the distribution. [Color figure can be viewed at wileyonlinelibrary.com]

Profiling for the Aβ, Tau, and αSyn biomarkers resulted in six groups of biomarker‐defined disease statuses, which were classified by their presumed underlying neurobiologically defined disease entity as follows (see Fig. 1, Table 1):

- Aβ^−^, Tau^−^, and αSyn^−^ profile: unclassified CBS;
- Aβ^+^, Tau^+^, and αSyn^−^ profile: AD;
- Aβ^−^, Tau^+^, and αSyn^−^ profile: tau‐predominant, reflecting that [^18^F]PI‐2620 uptake is most consistent with 4R tauopathy but does not allow definitive distinction from mixed 3R/4R tau;
- Aβ^−^, Tau^−^, and αSyn^+^ profile: Lewy‐type synucleinopathy (LTS);
- Aβ^+^, Tau^+^, and αSyn^+^ profile: co‐occurrence of AD and LTS;
- Aβ^−^, Tau^+^, and αSyn^+^ profile: co‐occurrence of tau‐predominant and LTS.

**TABLE 1:** Group demographics at baseline

	50 CBS split into:	Single protein biomarker status					
		50 CBS split into:		50 CBS split into:		50 CBS split into:	
	All	Aβ^+^	Aβ^−^	Tau^+^	Tau^−^	αSyn^+^	αSyn^−^
Stratification by		Positive ^18^F‐flutemetamol β‐amyloid‐PET (N = 8/11) and/or CSF β‐amyloid ratio 42/40 < 5.5% (N = 14/14)	Negative ^18^F‐flutemetamol β‐amyloid‐PET (N = 29/29) and/or CSF β‐amyloid ratio 42/40 ≥ 5.5% (N = 31/31)	Positive ^18^F‐PI‐2620 tau‐PET	Negative ^18^F‐PI‐2620 tau‐PET	Positive SAA aggregation curves	Negative SAA aggregation curves
Diagnostic certainty of clinical CBS phenotype (MDS‐PSP criteria)	s.o. PSP‐CBS n = 14; possible PSP‐CBS n = 36	s.o. PSP‐CBS n = 7; possible PSP‐CBS n = 7	s.o. PSP‐CBS n = 7; possible PSP‐CBS n = 29	s.o. PSP‐CBS n = 10; possible PSP‐CBS n = 35	s.o. PSP‐CBS n = 4; possible PSP‐CBS n = 1	s.o. PSP‐CBS n = 6; possible PSP‐CBS n = 6	s.o. PSP‐CBS n = 8; possible PSP‐CBS n = 30
Diagnostic certainty of clinical CBS phenotype (Armstrong CBD‐criteria)	Possible CBD‐CBS n = 21; probable CBD‐CBS n = 29	Possible CBD‐CBS n = 7; probable CBD‐CBS n = 6	Possible CBD‐CBS n = 14; probable CBD‐CBS n = 23	Possible CBD‐CBS n = 17; probable CBD‐CBS n = 28	Possible CBD‐CBS n = 4; probable CBD‐CBS n = 1	Possible CBD‐CBS n = 7; probable CBD‐CBS n = 5	Possible CBD‐CBS n = 14; probable CBD‐CBS n = 24
N_Baseline_	50	14 (28%)	36 (72%)	45 (90%)	5 (10%)	12 (24%)	38 (76%)
N_Longitudinal_	24	6 (25%)	18 (75%)	21 (87.5%)	3 (12.5%)	8 (33%)	16 (67%)
Sex (M/F)	24/26	8/6	16/20	23/22	1/4	7/5	17/21
Age (years)	71.3 ± 5.9	74.0 ± 5.7 *	70.0 ± 5.9 *	71.4 ± 5.8	69.1 ± 8.7	75.0 ± 6.5 *	69.9 ± 5.4 *
Disease duration (months)	37.6 ± 22.0	33.1 ± 27.3	39.0 ± 21.7	37.8 ± 23.5	33.4 ± 23.5	33.3 ± 20.2	38.6 ± 24.3
PSPRS	26.8 ± 13.7	25.9 ± 10.7	28.1 ± 14.7	29.1 ± 13.6	15.4 ± 7.6	19.4 ± 10.7*	29.9 ± 13.7*
MoCA	20.1 ± 6.3	15.9 ± 6.4**	23.0 ± 4.7**	20.9 ± 6.1	24.5 ± 3.3	21.1 ± 7.0	21.3 ± 5.7
DATE	33.4 ± 13.3	29.5 ± 13.2*	39.5 ± 11.6*	36.5 ± 12.8	41.5 ± 11.4	42.3 ± 8.2	35.6 ± 13.3
NfL (pg/ml)	2510.9 ± 1995.2	1958.1 ± 728.0	3354.0 ± 2370.2	2860.5 ± 2101.4	3410.0 ± 2036.5	1696.3 ± 803.9*	3299.9 ± 2226.2*
Aβ_42/40_ ratio (%)	6.7 ± 2.8	4.2 ± 0.8***	8.7 ± 3.5***	7.2 ± 3.8	7.7 ± 1.1	6.3 ± 1.7	7.6 ± 4.0

	Biomarker‐defined disease status					
	50 CBS split into:					
	Un‐classified	AD	Tau‐pred	LTS	AD+LTS	Tau‐pred+LTS
Stratification by	Aβ^−^ Tau^−^ Syn^−^	Aβ^+^ Tau^+^ Syn^−^	Aβ^−^ Tau^+^ Syn^−^	Aβ^−^ Tau^−^ Syn^+^	Aβ^+^ Tau^+^ Syn^+^	Aβ^−^ Tau^+^ Syn^+^
Diagnostic certainty of clinical CBS phenotype (MDS‐PSP criteria)	s.o. PSP‐CBS n = 2; possible PSP‐CBS n = 1	s.o. PSP‐CBS n = 5; possible PSP‐CBS n = 4	s.o. PSP‐CBS n = 1; possible PSP‐CBS n = 25	s.o. PSP‐CBS n = 2	s.o. PSP‐CBS n = 2; possible PSP‐CBS n = 3	s.o. PSP‐CBS n = 2; possible PSP‐CBS n = 3
Diagnostic certainty of clinical CBS phenotype (Armstrong CBD‐criteria)	Possible CBD‐CBS n = 2; probable CBD‐CBS n = 1	Possible CBD‐CBS n = 6; probable CBD‐CBS n = 3	Possible CBD‐CBS n = 6; probable CBD‐CBS n = 20	Possible CBD‐CBS n = 2	Possible CBD‐CBS n = 2; probable CBD‐CBS n = 3	Possible CBD‐CBS n = 3; probable CBD‐CBS n = 2
N_Baseline_	3 (6%)	9 (18%)	26 (52%)	2 (4%)	5 (10%)	5 (10%)
N_Longitudinal_	2 (8%)	3 (12.5%)	11 (46%)	1 (4%)	3 (12.5%)	4 (17%)
Sex (M/F)	1/2	5/4	11/15	0/2	3/2	4/1
Age (years)	67.3 ± 7.0	73.2 ± 4.9	69.1 ± 5.1	71.5 ± 13.4	75.4 ± 7.4	76.0 ± 2.9
Disease duration (months)	34.0 ± 33.0	31.8 ± 30.9	41.5 ± 21.3	32.5 ± 4.9	35.4 ± 22.6	31.6 ± 24.3
PSPRS	18.7 ± 7.4	27.6 ± 9.0	32.0 ± 15.1	10.5 ± 6.4	21.3 ± 15.5	21.8 ± 8.9
MoCA	23.0 ± 1.7	15.9 ± 6.6*	22.5 ± 5.0 *	29.0 ± 0.0	16.0 ± 6.8	24.6 ± 3.6
DATE	36.0 ± 3.6	26.1 ± 14.1	41.6 ± 7.3	58.0 ± 0.0 (n = 1)	38.3 ± 4.0	41.6 ± 7.3
NfL (pg/ml)	3410.0 ± 2036.5	2290.0 ± 738.8	3713.5 ± 2575.1	NA	1427.0 ± 248.6	1965.6 ± 1100.4
Aβ_42/40_ ratio (%)	8.1 ± 1.1	4.0 ± 0.8*	9.2 ± 4.2*	7.0 ± 0.8	4.5 ± 0.7	7.8 ± 0.9

*Note*: All results are displayed as mean (standard deviation) unless stated otherwise. Significance levels are derived from two‐sided Student‘s *t*‐tests for age and disease duration, significance levels for sex are derived from chi‐square tests (*P < 0.05, **P < 0.01, ***P < 0.001). For clinical and CSF parameters, significance levels of the logistic regression corrected for age, sex, and disease duration are shown in Table S1. Due to the relatively small group sizes, ANCOVA models for biomarker‐defined disease status groups corrected for age, sex, and disease duration yielded mostly insignificant *P*‐values, except for a significantly lower amyloid‐β_42/40_ ratio and MoCA in AD compared with the tau‐predominant group (*P* = 0.01 and *P* = 0.035, respectively, Tukey‘s post‐hoc test).

Abbrevations: CBS, corticobasal syndrome; Aβ^+^, amyloid‐β‐positive; Aβ^−^, amyloid‐β‐negative; Tau^+^, tau‐positive; Tau^−^, tau‐negative; tau‐pred, tau‐predominant pathology; αSyn^+^, α‐synuclein‐positive; αSyn^−^, α‐synuclein‐negative; AD, Alzheimer's disease; LTS, Lewy‐type synucleinopathy; PET, positron emission tomography; CSF, cerebrospinal fluid; SAA, seed amplification assay; s.o., suggestive of; PSP, progressive supranuclear palsy; CBD, corticobasal degeneration; MDS, International Parkinson and Movement Disorder Society; M, male; F, female; PSPRS, Progressive Supranuclear Palsy Rating Scale (higher scores indicate more severe impairment); MoCA, Montreal Cognitive Assessment (higher scores indicate better cognitive performance); DATE, Dementia Apraxia Test (higher scores indicate better performance); NfL, neurofilament light chain; NA, not assessed.

## Statistical Analysis

Statistical analyses were performed in R^34^ version 4.1.1 and Python^35^ version 3.9.18. Alpha thresholds were set to 0.05 for statistical significance testing. Sex ratios were compared by chi‐square tests. All continuous variables were tested for normality using the Shapiro–Wilk test (all *P* > 0.05) before application of two‐sided Student's *t*‐tests. To adjust for potential confounding factors, clinical and CSF parameters were then subject to logistic regression models corrected for age, sex, and disease duration to assess the effect of dichotomous biomarker statuses (Aβ, Tau, and αSyn). The hypothesis‐driven tests were restricted to five prespecified outcomes (CSF NfL, CSF Aβ42/40, PSPRS, MoCA, DATE); no formal multiplicity correction was applied. Exploratory analyses such as PSPRS subscores are displayed graphically without statistical inference. Next, the six observed disease statuses were subject to ANCOVAs with Tukey's post‐hoc test corrected for age, sex, and disease duration. Error bars indicate the standard deviation (SD). In a linear mixed‐effects model (lmer package), the interaction effect of each baseline biomarker (ie, Tau, Aβ, and αSyn) with time on PSPRS was modeled to test whether the biomarkers would predict on the clinical trajectories in a subset of patients with clinical follow‐up visits. The model was controlled for age, sex, disease duration, number of follow‐up visits per subject, and random slope and intercept.

## Results

### Study Cohort

Fifty‐eight CBS patients met the inclusion criteria^1^, ^2^ and provided written informed consent. Two patients tested positive for rare pathogenic variants in the *LRRK2* and *GBA1* genes, respectively, and were excluded from analysis. Six further patients were excluded from analysis due to incomplete assessment of all three biomarker categories (Aβ, Tau, and αSyn). In total, 50 CBS patients with complete biomarker status were included into the analysis.

### Frequency of Aβ, Tau, and αSyn Biomarkers in CBS

Among the 50 CBS patients, 14 (28%) were Aβ^+^, 45 (90%) were Tau^+^, and 12 (24%) were αSyn^+^ (Fig. 1). Of note, numerous patients were positive for more than one biomarker (eg, all Aβ^+^ CBS patients were also Tau^+^). Detailed demographic and clinical data of these subgroups are presented in Table 1. To study the effect of each individual biomarker status on the manifestation of CBS, we first split the cohort dichotomously by positivity or negativity for single biomarkers, regardless of the other biomarkers.

Aβ^+^ and Aβ^−^ CBS patients had no significant differences in sex distribution or disease duration, but Aβ^+^ CBS patients were significantly older at the time of examination than Aβ^−^ patients (74.0 ± 5.7 vs. 70.0 ± 5.9 years; *P* < 0.05).

Tau^+^ and Tau^−^ CBS patients had no significant differences regarding sex distribution, disease duration, and age at examination, although these analyses were limited by the small numbers (N = 5) of Tau^−^ patients.

αSyn^+^ and αSyn^−^ CBS patients had no significant differences regarding disease duration or sex distribution, but αSyn^+^ patients were significantly older at examination than αSyn^−^ patients (75.0 ± 6.5 vs. 69.9 ± 5.4 years; *P* < 0.05).

### Frequency of Biomarker‐Defined Disease Statuses in CBS

Next, we classified the patients into six groups of biomarker‐defined disease status, based on their presumed underlying neurobiologically defined disease entity (Fig. 1). The order of classification (ie, starting either by Aβ, Tau, or αSyn) naturally did not affect the allocation of the individual patients into their final disease status group (Fig. S1).

While only 3 (6%) remained unclassified (Aβ^−^, Tau^−^, αSyn^−^), 9 (18%) were classified as having AD (Aβ^+^, Tau^+^, and αSyn^−^), 26 (52%) as tau‐predominant (Aβ^−^, Tau^+^, αSyn^−^), 2 (4%) as LTS (Aβ^−^, Tau^−^, αSyn^+^), 5 (10%) as AD+LTS (Aβ^+^, Tau^+^, αSyn^+^), and 5 (10%) as tau‐predominant+LTS (Aβ^−^, Tau^+^, αSyn^+^) (Table 1, Fig. 1). Detailed demographic and clinical data did not differ between these subgroups (Table 1). The tau‐PET visual read showed 100% concordance with a typical 3R/4R tauopathy binding pattern in Aβ^+^/Tau^+^ patients, whereas Aβ^−^/Tau^+^ individuals consistently lacked such a pattern (Fig. S2).

### Effect of the Aβ, Tau, and αSyn Biomarker Status on NfL and Aβ Levels

**FIGURE. 2:**
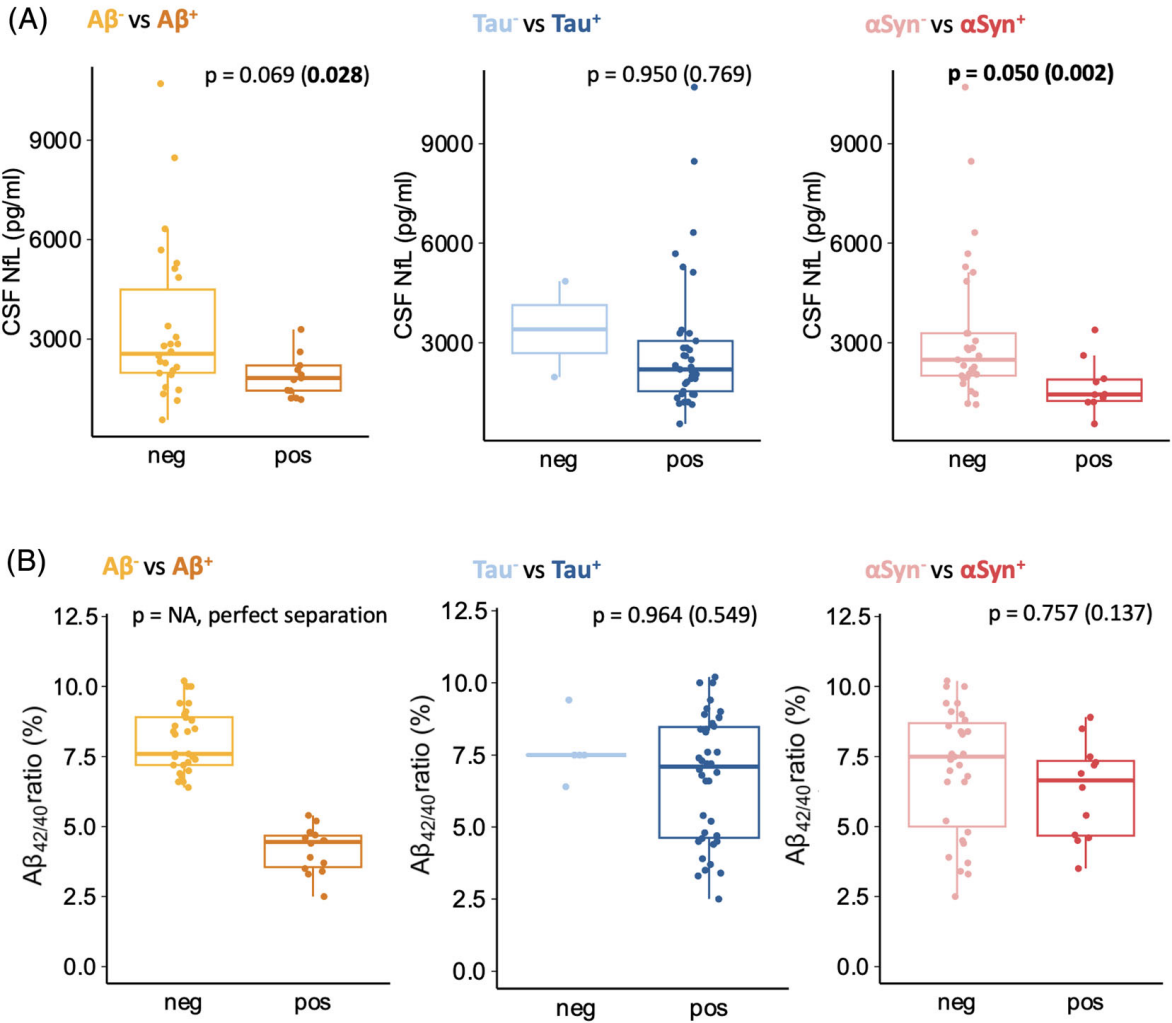
Association between the biomarker status (Aβ, Tau, and αSyn) and other cerebrospinal fluid (CSF) biomarkers. Aβ^+^, amyloid‐β‐positive; Aβ^−^, amyloid‐β‐negative; αSyn^+^, α‐synuclein‐positive; αSyn^−^, α‐synuclein‐negative; Tau^+^, tau‐positive; Tau^−^, tau‐negative. The boxplots visualize the distribution of neurofilament light chain (NfL) values (A) and amyloid‐*β*_42/40_ ratio values (B) across Aβ^+^ and Aβ^−^, or Tau^+^ and Tau^−^, or αSyn^+^ and αSyn^−^ groups. The *P*‐values displayed are derived from the logistic regression model, which tests the association between amyloid‐β_42/40_ ratio or NfL and Aβ, Tau, or αSyn status while controlling for age, disease duration, and sex. *P*‐values in brackets are derived from single‐variate statistics employing two‐sided Student's *t*‐tests after checking for normality (Shapiro–Wilk test). [Color figure can be viewed at wileyonlinelibrary.com]

We then investigated the association of the Aβ, Tau, and αSyn biomarker status with the CSF biomarkers NfL and Aβ42/40. Figure 2 displays boxplots of NfL levels (A) and Aβ_42/40_ ratios (B) in subgroups stratified by their Aβ, Tau, and αSyn status. We first performed single‐variable analyses (Student's *t*‐tests) to explore unadjusted relationships between biomarker status and NfL levels and Aβ42/40 ratios. Next, we applied logistic regression models to account for potential confounding factors, including age, disease duration, and sex (Table S1).

NfL levels (Fig. 2A) were lower in the Aβ^+^ versus the Aβ^−^ group by single‐variable analysis (*P* < 0.05), but the difference was no longer significant after adjusting for confounding factors by logistic regression. No significant differences in the NfL concentrations were observed between Tau^+^ versus Tau^−^ groups. αSyn^+^ patients had lower NfL levels compared with αSyn^−^ patients (both single‐variable‐ and regression‐derived *P* < 0.05). The significantly older age of αSyn^+^ CBS patients underscores the significance of this finding, because NfL levels are generally expected to increase with age.^36^

The Aβ_42/40_ ratio (Fig. 2B) perfectly separated Aβ^+^ from Aβ^−^ patients, as expected. No significant differences in the Aβ_42/40_ ratio were found for the Tau^+^ versus Tau^−^ or the αSyn^+^ versus αSyn^−^ groups.

### Effect of the Biomarker‐Defined Disease Status on NfL and Aβ Levels

We then investigated the association of the biomarker‐defined disease status with the CSF biomarkers NfL and Aβ_42/40_. Figure S3 displays boxplots of NfL levels (A) and Aβ_42/40_ ratios (B) in subgroups stratified by their disease status. NfL levels (Fig. S3A) were highest in the unclassified and in the tau‐predominant group, but not significantly different in the ANCOVA model. The Aβ_42/40_ ratio (Fig. S3B) expectedly was significantly lower in AD compared with tau‐predominant (*P* < 0.05, ANCOVA with Tukey's post‐hoc test).

### Effect of the Aβ, Tau, and αSyn Biomarker Status on Clinical Features

**FIGURE. 3:**
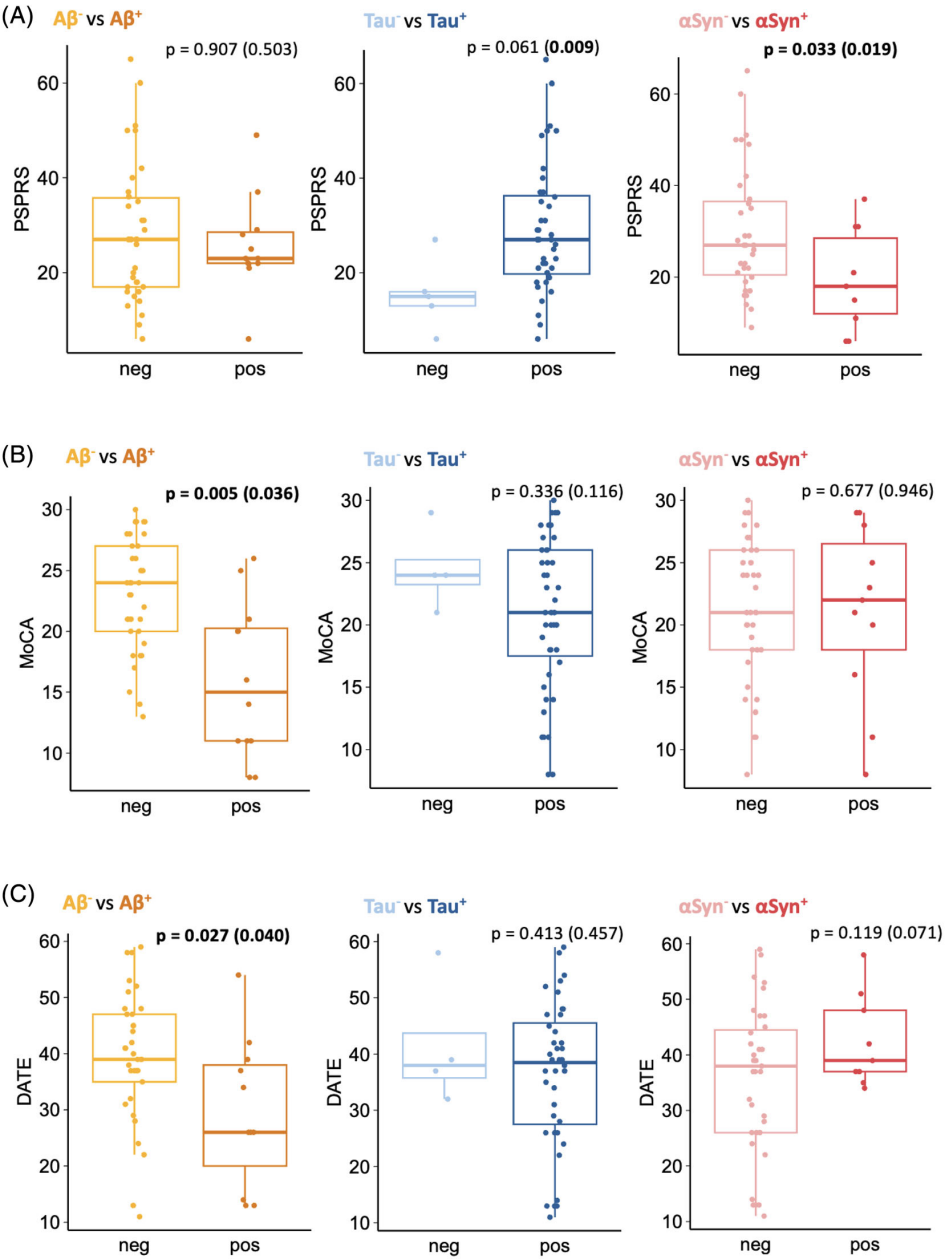
Association between the biomarker status (Aβ, Tau, and αSyn) and clinical measures of disease severity. Aβ^+^, amyloid‐β‐positive; Aβ^−^, amyloid‐β‐negative; αSyn^+^, α‐synuclein‐positive; αSyn^−^, α‐synuclein‐negative; Tau^+^, tau‐positive; Tau^−^, tau‐negative; PSPRS, Progressive Supranuclear Palsy Rating Scale (higher scores indicate more severe impairment); MoCA, Montreal Cognitive Assessment (higher scores indicate better cognitive performance); DATE, Dementia Apraxia Test (higher scores indicate better performance). The boxplots visualize the distribution of PSPRS (A), MoCA (B), and DATE (C) scores across Aβ^+^ and Aβ^−^, or Tau^+^ and Tau^−^, or αSyn^+^ and αSyn^−^ groups. The *P*‐value displayed is derived from the logistic regression model, which tests the association between PSPRS, MoCA, or DATE and Aβ, Tau, or αSyn status while controlling for age, disease duration, and sex. *P*‐values in brackets are derived from single‐variate statistics employing two‐sided Student's *t*‐tests after checking for normality (Shapiro–Wilk test). [Color figure can be viewed at wileyonlinelibrary.com]

We investigated the association of the Aβ, Tau, and αSyn biomarker status with clinical measures of disease severity. Figure 3 displays boxplots of PSPRS (**A**), MoCA (**B**), and DATE (**C**) scores in subgroups stratified by their Aβ, Tau, and αSyn status. We first performed single‐variable analyses to explore unadjusted relationships, followed by logistic regression models accounting for potential confounding factors (age, disease duration, and sex).

PSPRS scores (Fig. 3A) were not significantly different between the Aβ^+^ and Aβ^−^ groups.

Aβ^+^ CBS patients exhibited significantly lower MoCA scores (Fig. 3B; Table S1; single‐variable‐derived: *P* < 0.05, regression‐derived: *P* < 0.01) and DATE scores (Fig. 3C; Table S1; both single‐variable‐ and regression‐derived: *P* < 0.05) compared with Aβ^−^ patients. These findings indicate greater cognitive impairment in Aβ^+^ cases.

The Tau status had no significant associations with PSPRS, MoCA, or DATE scores (Fig. 3A–C, Table S1), although PSPRS scores trended higher in Tau^+^ patients (single‐variable‐derived: *P* < 0.01, regression‐derived: *P* = 0.061), suggesting a potential influence on motor severity that warrants further study.

Interestingly, the αSyn^+^ status was associated with lower PSPRS scores (Fig. 3A, Table S1; both single‐variable‐ and regression‐derived: *P* < 0.05). These observations suggest an association between αSyn‐positivity and relatively milder motor symptoms. MoCA and DATE scores (Fig. 3B,C) did not differ significantly between the αSyn^+^ and αSyn^−^ groups. Figure S4 provides descriptive boxplots of the individual PSPRS subscores.

### Effect of the Biomarker‐Defined Disease Status on Clinical Features

We investigated the association of the biomarker‐defined disease status with these clinical measures of disease severity (Fig. S5). The ANCOVA models demonstrated significantly lower (ie, worse) MoCA scores in the AD group compared with the tau‐predominant group (*P* < 0.05; Fig. S5B), as well as the following non‐significant trends:

The mean PSPRS scores (Fig. S5A) were highest (ie, worst) in the tau‐predominant group and lowest (ie, best) in the LTS group. The mean MoCA scores (Fig. S5B) were lowest (ie, worst) in the AD and AD+LTS groups and highest (ie, best) in the LTS group. The DATE scores (Fig. S3C) were lowest (ie, worst) in the AD group and highest (ie, best) in the LTS group. Due to the relatively small group sizes, ANCOVA models corrected for age, sex, and disease duration yielded insignificant *P*‐values for all other comparisons of the biomarker‐defined disease groups.

We next analyzed the presence of symptoms typically associated with LTS, as observed in Parkinson's disease or dementia with Lewy bodies, including resting tremor, restless legs syndrome (RLS), postural tremor, orthostatic symptoms, visual hallucinations, and hyposmia (Fig. S6). LTS‐typical clinical features were more prevalent in αSyn^+^ patients versus αSyn^−^ patients; however, in the Fisher exact test, no significant differences between the different biomarker and disease statuses were observed.

### Effect of Biomarker and Disease Status on Longitudinal Changes in PSPRS and NfL

Longitudinal data were available for 24 of the 50 CBS patients, some of which have been reported previously.^37^ Their biomarker status is reported in Table 1. The median clinical follow‐up period was 1.9 years, ranging from 0.8 to 4.1 years. No changes in the clinical diagnosis of CBS were observed at follow‐up visits. We observed a trend toward worse motor status in subjects without follow‐up visits compared with those with follow‐up (*t*‐test: PSPRS = 31.7 ± 13.6 vs. PSPRS = 23.9 ± 13.1; *P* = 0.06), as well as worse cognitive status (MoCA = 19.5 ± 5.8 vs. MoCA = 22.9 ± 5.7; *P* = 0.05). This suggests that patients with more severe clinical status are less likely to attend follow‐up visits. Despite this potential selection bias, the follow‐up time had a significant effect on PSPRS scores with an annualized increase of 7.0 ± 0.9 points (*P* < 0.001) in the linear mixed‐effects model, confirming progressive clinical deterioration in the longitudinal CBS cohort. The random slope‐intercept correlation of 0.41 suggests a moderate association, indicating that patients with higher PSPRS scores at baseline tend to experience a faster rate of decline.

For 15 CBS patients, at least two NfL measurements in CSF were available. Follow‐up time had a significant effect on NfL levels, with an annualized worsening of 665.5 ± 245.4 (*P* < 0.05) in an adjusted model. Again, a random slope‐intercept correlation of 0.47 suggests that patients with higher NfL levels at baseline tend to show a more rapid increase in NfL levels over time.

**FIGURE. 4:**
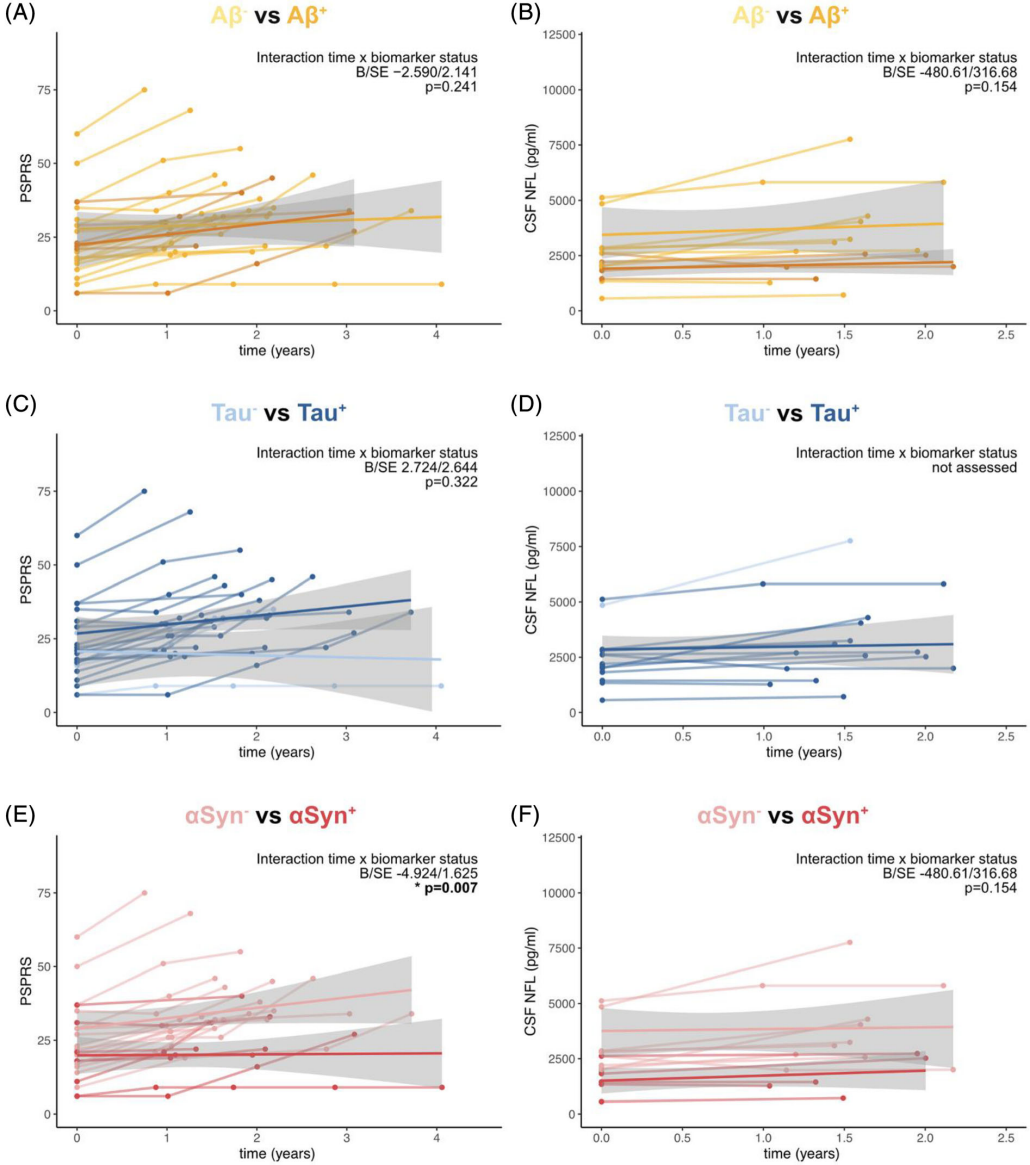
Interaction of the biomarker status (Aβ, Tau, and αSyn) and disease progression. Aβ^+^, amyloid‐β‐positive; Aβ^−^, amyloid‐β‐negative; αSyn^+^, α‐synuclein‐positive; αSyn^−^, α‐synuclein‐negative; Tau^+^, tau‐positive; Tau^−^, tau‐negative; PSPRS, Progressive Supranuclear Palsy Rating Scale (higher scores indicate more severe impairment); NfL, neurofilament light chain. Line plots illustrating clinical trajectories on the PSPRS (A, C, E), and NfL (B, D, F) in the subset of patients with longitudinal visits stratified by Aβ, Tau, and αSyn status. Linear model fits (ie, least squares line) are indicated together with 95% confidence intervals. Statistics are based on linear mixed models controlling for age, sex, disease duration, numbers of follow‐up visits per patient, and random slope and intercept. For visualization, regression fits were split into dichotomous biomarker status to illustrated disease trajectories relative to biomarker abnormality; however, interactions were computed using continuous measures. [Color figure can be viewed at wileyonlinelibrary.com]

We then examined whether the biomarker status would predict the rate of disease progression, measured by the clinical PSPRS scale or NfL levels, using linear mixed‐effects models adjusted for age, sex, disease duration, numbers of follow‐up visits per patient, and random slope and intercept (Fig. 4). The Aβ status had no significant interaction with time for PSPRS (Fig. 4A) or NfL levels (Fig. 4B). The Tau status had no significant effect on PSPRS progression (Fig. 4C) and could not be analyzed for NfL due to limited longitudinal data in Tau^−^ CBS patients (Fig. 4D). The αSyn status showed significant interaction with time for the outcome variable PSPRS (*P* < 0.05; Fig. 4E), indicating that αSyn^−^ CBS patients in our cohort progressed faster than αSyn^+^ patients. The estimated annualized increase of the PSPRS for αSyn^−^ CBS subjects was 8.5 ± 1.8, while it was 3.6 ± 5.1 for αSyn^+^ CBS subjects. However, no significant effect was found for NfL (Fig. 4F).

In our cohort, CBS patients with presumed tau‐predominant pathology experienced faster increases in (ie, worsening of) PSPRS score compared with tau‐predominant with LTS co‐pathology (*P* < 0.05; Fig. S7A,B), but not of NfL levels over time (Fig. S7C); however, the small sample size of tau‐predominant+LTS (n = 4) should be considered when interpreting these results.

## Discussion

This study represents a first attempt to classify CBS patients in a prospectively studied cohort based on biomarker profiles. Our study classified CBS patients based on single protein biomarkers (Aβ, Tau, and αSyn) and secondly the biomarker‐defined disease status (AD, tau‐predominant, LTS, mixed pathologies, and unclassified patients) on the cross‐sectional and longitudinal clinical manifestations of CBS. The distribution in our cohort of presumed pathologies is similar to histopathological distribution of CBS patients in autopsy‐confirmed cases.^4^, ^6^, ^7^ The single protein biomarkers Aβ, Tau, and αSyn identified presumed underlying pathologies in 94% (47/50) of CBS patients. Some 6% of the CBS cases (3/50) remained “unclassified” with negative Aβ, Tau, and αSyn status, likely due to other neuropathologies, (eg, TDP‐43 proteinopathy). αSyn‐positivity in isolation classifying as LTS (Aβ^−^, Tau^−^, αSyn^+^) was quite rare (n = 2/50, 4%), but more common in combination with other pathologies, particularly in CBS cases with presumed AD (AD+LTS; n = 5/14, 36%), compared with tau‐predominant pathology (tau‐predominant+LTS; n = 5/31, 16%).

While FDG‐PET is a valuable imaging biomarker for assessing neuronal dysfunction and has demonstrated utility in distinguishing CBS due to AD from CBS without AD^38^, our study followed a conceptually different approach by classifying CBS patients based on biomarkers that more directly reflect the presumed histopathology. Regarding the stratification of Tau^+^ and Tau^−^ using [^18^F]PI‐2620 tau‐PET as an imaging biomarker for tau aggregation in vivo, multiple lines of evidence from preclinical and clinical studies by independent groups support the use of [^18^F]PI‐2620 tau‐PET for detecting 4R tau pathology.^13^, ^14^, ^15^, ^18^, ^20^, ^39^, ^40^, ^41^ This includes consistent binding to 4R tau aggregates in vitro, translational correlation with in vivo PET signals in animal models and human tissue, and validated discrimination of 4R tauopathies from AD and healthy controls in clinical imaging studies. In the current study, the tau‐PET visual read showed 100% concordance with a typical 3R/4R tauopathy binding pattern in Aβ^+^/Tau^+^ patients, whereas Aβ^−^/Tau^+^ individuals consistently lacked such a pattern. This absence of a 3R/4R binding pattern, combined with predominant signal in the basal ganglia,^17^ further supported their classification as tau‐predominant CBS. Nevertheless, as [^18^F]PI‐2620 does not conclusively differentiate between 4R tau and mixed 3R/4R tau pathologies, we conservatively use the term “tau‐predominant” rather than “4R tauopathy.”

Aβ^+^ patients showed significantly greater cognitive impairment, with lower MoCA scores and DATE scores, compared with Aβ^−^ patients, highlighting a more pronounced cortical involvement in Aβ^+^ cases. While Tau^+^ CBS cases showed a trend toward higher PSPRS scores, no significant differences in disease progression rates were observed compared with Tau^−^ patients, likely due to small sample sizes. αSyn^+^ patients exhibited significantly lower PSPRS scores and a slower clinical progression over time. NfL levels were significantly lower in αSyn^+^ compared with αSyn^−^ patients, confirmed by both logistic regression and mixed‐effects models, and consistently observed in the tau‐predominant+LTS and AD+LTS subgroups. This suggests that αSyn positivity may act as a modulating factor in CBS, associated with a milder disease course and distinct symptom profile. However, it is important to note that our longitudinal analysis might underestimate disease progression in more severely affected patients, as these individuals are less likely to attend follow‐up visits.

The distribution of presumed pathologies in our cohort aligns closely with the histopathological distribution observed in autopsy‐confirmed CBS cases.^4^, ^5^, ^6^ In 4R tauopathies, there is limited evidence on the clinical impact of mixed pathologies. In histopathologically confirmed PSP, mixed pathologies are frequently observed in over 80% of cases, with focal AD‐related pathology being the most common co‐pathology.^42^ The presence of co‐pathologies did not significantly affect clinical milestones like disease duration in this study, but age of onset was younger in patients with argyrophilic grains. Between 8% and 31.5%^42^, ^43^, ^44^, ^45^, ^46^ of PSP cases exhibit both PSP and LTS. In pathologically confirmed CBD cases, LTS has been found in approximately 10%^47^ to 14.3%^48^ of cases. In these CBD cohorts, co‐pathologies such as Aβ deposition, cerebral amyloid angiopathy, and limbic‐predominant or age‐related TDP‐43 encephalopathy were also observed, particularly in older patients. However, clinical correlations specific to CBD with these co‐pathologies remain unreported. A case reported by Yamashita et al.^49^ describes a patient with an 18‐year disease duration until death who exhibited a co‐pathology of TDP‐43, αSyn, and CBD, suggesting that certain mixed pathologies in CBS may be associated with prolonged survival. A recent article investigated AD pathology in 35 cases of clinically diagnosed CBS based on the AD Neuropathological Change criteria.^50^ Postmortem AD pathology was identified in 40% of CBS cases, with 23% classified as the primary pathology – defined as the one most closely linked to the clinical syndrome. The remaining cases exhibited AD as a co‐pathology to 4RT or rarely TDP‐43 pathology. LTS pathology was absent in their CBS cohort.

The presence of Aβ and αSyn co‐pathologies raises critical questions regarding disease mechanisms, progression, and the temporal sequence of pathological events. Neuropathological studies provide insights into end‐stage disease but lack the capacity to clarify the sequential development of mixed pathologies. It remains unclear which pathology initiates disease progression or how the presence of one pathology influences the manifestation and progression of the other: it is unknown whether a hierarchical relationship exists, such as AD with secondary αSyn co‐pathology or, conversely, LTS with secondary AD features.

In first in vivo studies of AD populations, αSyn positivity has been linked to accelerated cognitive decline,^51^, ^52^ though its potential effects on motor symptoms in the context of AD remain unexplored. This raises the question of whether the coexistence of αSyn pathology exacerbates neurodegenerative processes through a synergistic effect, specifically in cognitive decline. Understanding the temporal order and possible hierarchical interactions between AD and αSyn pathologies is essential to delineate distinct clinical entities. Our data indicate that identifying these mixed pathologies in CBS could carry significant implications for prognosis and disease management, underscoring the need for targeted therapeutic strategies that address the specific pathologies involved.

Three studies have evaluated αSyn SAA in CBS, yielding varying prevalence estimates. Anastassiadis et al.^53^ found a higher αSyn positivity rate in Aβ^−^ CBS cases using a different assay (35.9% of patients with Aβ^−^ CBS and 28.6% with PSP). NfL levels in the αSyn^+^ subgroup were increased, in contrast to our findings. Second, in a retrospective cohort of UK PSP and CBS cases using the Amprion αSyn SAA,^54^ 46% (6/13) cases of AD‐CBS and 19% (3/16) of a cohort consisting of non‐AD‐CBS and CBS with unknown AD status were αSyn positive, yielding again a higher prevalence of mixed pathology than our cohort.^54^ In CBS, there was no clinical difference between αSyn‐positive and αSyn‐negative CBS. In PSP, exploratory analysis showed a trend toward αSyn^+^ PSP participants being older, more impaired in motor, cognitive, and functional scales. Baiardi et al^55^ analyzed a cohort of 29 CBS cases, of which 48% had a positive Aβ status in CSF, and only one αSyn‐positive case (3.4%) was reported, which is fewer than expected from histopathological studies.^55^ Tau biomarker status was not assessed in these three studies. Our study's strength lies in the availability of longitudinal data and comprehensive biomarker assessment for all three biomarkers of Aβ, Tau, and αSyn, allowing for a more nuanced exploration of the relationship between biomarker positivity and clinical outcomes in CBS.

Our study has several limitations. First, our cohort primarily reflects a Caucasian population, which may limit the generalizability of our findings. Second, although biomarkers provide valuable in vivo information, neuropathological confirmation remains the gold standard for diagnosis. A continued clinical follow‐up, with postmortem analysis where possible, will be essential to validate the sensitivity and specificity of Aβ, αSyn, and Tau biomarkers in CBS. Additionally, our study did not employ multiple testing corrections due to the limited sample size. Subanalyses involving the small Tau^−^ (n = 5) and αSyn ^+^ (n = 12) groups are underpowered; their nominal *P*‐values are presented without multiplicity correction and should be considered exploratory until confirmed in independent cohorts. Although our cohort is too small for a formal Tau × αSyn interaction test, recent in vivo evidence from AD shows that α‐synuclein co‐pathology accelerates amyloid‐driven tau accumulation,^56^ underlining the importance of addressing such interactions in CBS once larger, quantitatively‐imaged samples are available. Finally, αSyn SAA may also have limited sensitivity for early amygdala or olfactory bulb predominant LTS^21^, highlighting the need for αSyn PET imaging in the future that may also allow assessment of spatial distribution of αSyn. Future studies should aim to incorporate promising TDP‐43 pathology biomarkers^57^ to further classify unclassified CBS cases and assess other relevant proteinopathies.

Our study supports the notion that CBS is a polyetiological condition requiring molecular‐level subclassification to facilitate the development of targeted therapies. Previously, such subclassification was only achievable postmortem, but with recent biomarker advancements, it is now feasible in vivo. Biomarker‐based analysis reveals that Aβ^+^ CBS patients exhibit more cognitive symptoms, while αSyn^+^ CBS cases, particularly those with Aβ^−^ status, follow a milder clinical trajectory. This molecular stratification could inform personalized therapeutic strategies and planning of clinical trials in CBS.

## Author Roles

(1) Research Project: A. Concepualization, B. Resources, C. Methodology, D. Investigation, E. Data Curation, F. Formal Analysis; (2) Statistical Analysis: A. Design, B. Execution, C. Review and Critique; (3) Manuscript Preparation: A. Writing the Original Draft, B. Review and Editing; (4) A. Visualization, B. Project Administration (Ethics Approvals), C. Recruitment and Clinical Characterization of Patients' Cerebrospinal Fluid, D. Performing Seed Amplification Assay Procedures, E. Supervision, F. Funding Acquisition.

C.P.: 1A, 1B, 1C, 1D, 1E, 1F, 3A, 3B, 4A, 4B, 4C.

A.M.B.: 1A, 1B, 1C, 1D, 1E, 1F, 3A, 3B, 4A, 4B, 4C, 4D.

E.W.: 1A, 1C, 1D, 3B, 4C,

U.M.F.: 3B, 4C.

A.J.: 3B, 4C.

S.K.: 3B, 4C.

J.G.: 1B, 3B.

T.B.: 1B, 1D, 3B.

N.F.: 1F, 3B.

R.P.: 1B, 3B.

M.B.: 1A (lead), 1B, 3B, 4E (lead), 4F (lead).

J.L.: 1A (lead), 1C (lead), 3B, 4E (lead), 4F (lead).

G.U.H.: 1A (lead), 1C (lead), 3B, 4E (lead), 4F (lead).

## Funding

The recruitment of the CBS cohort was supported by the presidential fund of the Helmholtz Society. This project was also supported by the German Center for Neurodegenerative Diseases (DZNE, DescribePSP Study), the German Parkinson's Association (DPG, ProPSP Study), and the Hirnliga e.V. (Manfred‐Strohscheer‐Stiftung). Tau‐PET imaging was funded by the Alzheimer Forschung Initiative e.V. (grant number #19063p). M.B.'s work was funded by the Deutsche Forschungsgemeinschaft (DFG, German Research Foundation; project numbers BR4580/1‐1/RO5194/1‐1). C.P., G.U.H, J.L., and M.B. were supported by the Deutsche Forschungsgemeinschaft (DFG, German Research Foundation) under Germany's Excellence Strategy within the framework of the Munich Cluster for Systems Neurology (EXC 2145 SyNergy: ID 390857198). J.L. was supported by the German Ministry of Research and Education (Förderkennzeichen: FKZ161L0214B, FKZ161L0214C CLINSPECT‐M). G.U.H. was also funded by the NOMIS Foundation (FTLD project), Volkswagen Stiftung/Lower Saxony Ministry for Science/Petermax‐Müller Foundation (Etiology and Therapy of Synucleinopathies and Tauopathies), European Joint Programme on Rare Diseases (Improve‐PSP). The Lüneburg Heritage has supported the work of C.P., S.K., and J.L. C.P. was also funded by Thiemann Stiftung and Else‐Kröner‐Fresenius‐Stiftung. A.M.B. and J.L. receive funding from The Michael J. Fox Foundation (Alpha‐Synuclein Posttranslational Modifications Program).

## Conflicts of Interest

C.P. are J. L. are inventors in a patent “Oral Phenylbutyrate for Treatment of Human 4‐Repeat Tauopathies” (PCT/EP2024/053388) filed by LMU Munich. A.M.B. and J.L. are inventors of a patent application related to quantification of α‐synuclein aggregates using SAAs. N.F. received consulting honoraria from MSD, speaker honoraria from EISAI, GE Healthcare and Life Molecular Imaging as well as research support from Eli Lilly. M.B. has received speaker honoraria from Roche, GE Healthcare, and Life Molecular Imaging; has advised Life Molecular Imaging; and is currently on the advisory board of MIAC. J.L. reports speaker fees from Bayer Vital, Biogen, EISAI, TEVA, Zambon, Esteve, Merck and Roche, consulting fees from Axon Neuroscience, EISAI and Biogen, author fees from Thieme medical publishers and W. Kohlhammer GmbH medical publishers. In addition, he reports compensation for serving as chief medical officer for MODAG GmbH, is beneficiary of the phantom share program of MODAG GmbH and is inventor in a patent “Pharmaceutical Composition and Methods of Use” (EP 22159408.8) filed by MODAG GmbH, all activities outside the submitted work. G.U.H. participated in industry‐sponsored research projects from AbbVie, Biogen, Biohaven, Novartis, Roche, Sanofi, and UCB; has ongoing research collaborations with Roche, UCB, and AbbVie; serves as a consultant for AbbVie, Alzprotect, Amylyx, Aprineua, Asceneuron, Bayer, Bial, Biogen, Biohaven, Epidarex, Ferrer, Kyowa, Kirin, Lundbeck, Novartis, Retrotope, Roche, Sanofi, Servier, Takeda, TEVA, and UCB; received honoraria for scientific presentations from AbbVie, Bayer, Bial, Biogen, Bristol Myers Squibb, Kyowa Kirin, Pfizer, Roche, TEVA, UCB, and Zambon.

## Supporting information


**Figure S1.** Biomarker‐guided classification to corticobasal syndrome patients.
**Figure S2.** Representative [^18^F]PI‐2620 tau‐PET (positron emission tomography) standardized uptake value ratio (SUVR) images across biomarker‐defined corticobasal syndrome subgroups.
**Figure S3.** Association between the presumed biomarker‐defined disease status and selected cerebrospinal fluid biomarkers.
**Figure S4.** Association between the biomarker status (Aβ, Tau, and αSyn) and Progressive Supranuclear Palsy Rating Scale (PSPRS) subscores.
**Figure S5.** Association biomarker‐defined disease status and clinical scores (Progressive Supranuclear Palsy Rating Scale [PSPRS], Montreal Cognitive Assessment [MoCA], Dementia Apraxia Test [DATE]).
**Figure S6.** Presence of clinical features in biomarker‐based subgroups of corticobasal syndrome.
**Figure S7.** Interaction of the biomarker‐defined disease status and disease progression.

**Table S1.** Logistic regression analysis of Aβ, Tau, and αSyn status on disease severity and biomarkers.

## Data Availability

The data that support the findings of this study are available on request from the corresponding author. The data are not publicly available due to privacy or ethical restrictions.
