# Supplementary material for: A Biomarker‐Based Classification of Corticobasal Syndrome
Source: Mov Disord. 2025 Oct 6;41(1):129–42. doi: 10.1002/mds.70070 (PMC12882055; doi:10.1002/mds.70070)
Supplement: Supplementary file 1 — Figure S1. Biomarker‐guided classification to corticobasal syndrome patients. Figure S2. Representative [18F]PI‐2620 tau‐PET (positron emission tomography) standardized uptake value ratio (SUVR) images across biomarker‐defined corticobasal syndrome subgroups. Figure S3. Association between the presumed biomarker‐defined disease status and selected cerebrospinal fluid biomarkers. Figure S4. Association between the biomarker status (Aβ, Tau, and αSyn) and Progressive Supranuclear Palsy Rating Scale (PSPRS) subscores. Figure S5. Association biomarker‐defined disease status and clinical scores (Progressive Supranuclear Palsy Rating Scale [PSPRS], Montreal Cognitive Assessment [MoCA], Dementia Apraxia Test [DATE]). Figure S6. Presence of clinical features in biomarker‐based subgroups of corticobasal syndrome. Figure S7. Interaction of the biomarker‐defined disease status and disease progression. [file MDS-41-129-s002.pdf]

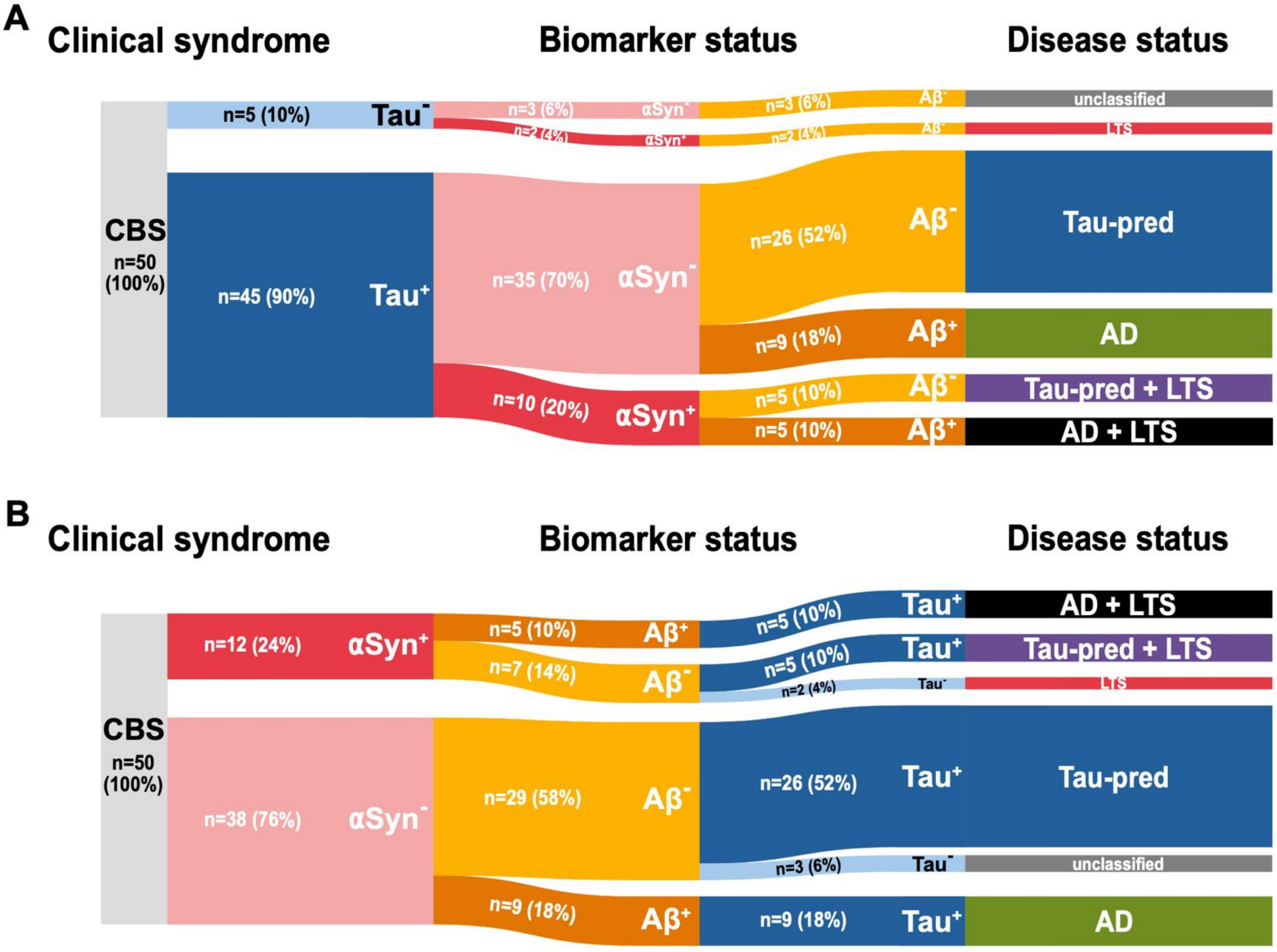

Supplementary Figure 1: Biomarker guided classification of corticobasal syndrome patients

Aβ+: Amyloid-β-positive, Aβ-: Amyloid-β-negative, αSyn+: α-Synuclein-positive, αSyn-: α-Synuclein-negative, Tau+: Tau-positive, Tau-: Tau-negative, tau-pred: tau-predominant pathology, AD: Alzheimer's Disease pathology, LTS: Lewy-type Synucleinopathy.

This figure presents alternative, unbiased biomarker-guided approaches to classify corticobasal syndrome (CBS) patients into six distinct groups based on Aβ, Tau, and αSyn status. Panel A shows an approach starting with Tau stratification, followed by αSyn, and then Aβ. Panel B starts with αSyn stratification, followed by Aβ, and then Tau. In both cases, patients are categorized based on their biomarker status: Patients were screened for Aβ by Amyloid-PET or CSF and denoted as Aβ- in light yellow or Aβ+ in dark yellow if one of these measurements showed pathological results. Tau-PET was employed to stratify patients into Tau- in light blue and Tau+ in dark blue. αSyn seed amplification assay from CSF was employed to categorize patients into αSyn- and αSyn+ displayed in light red and dark red, respectively. Despite varying the order of biomarker stratification, the resulting classification consistently identifies the same six groups, each representing a distinct presumed underlying pathology.

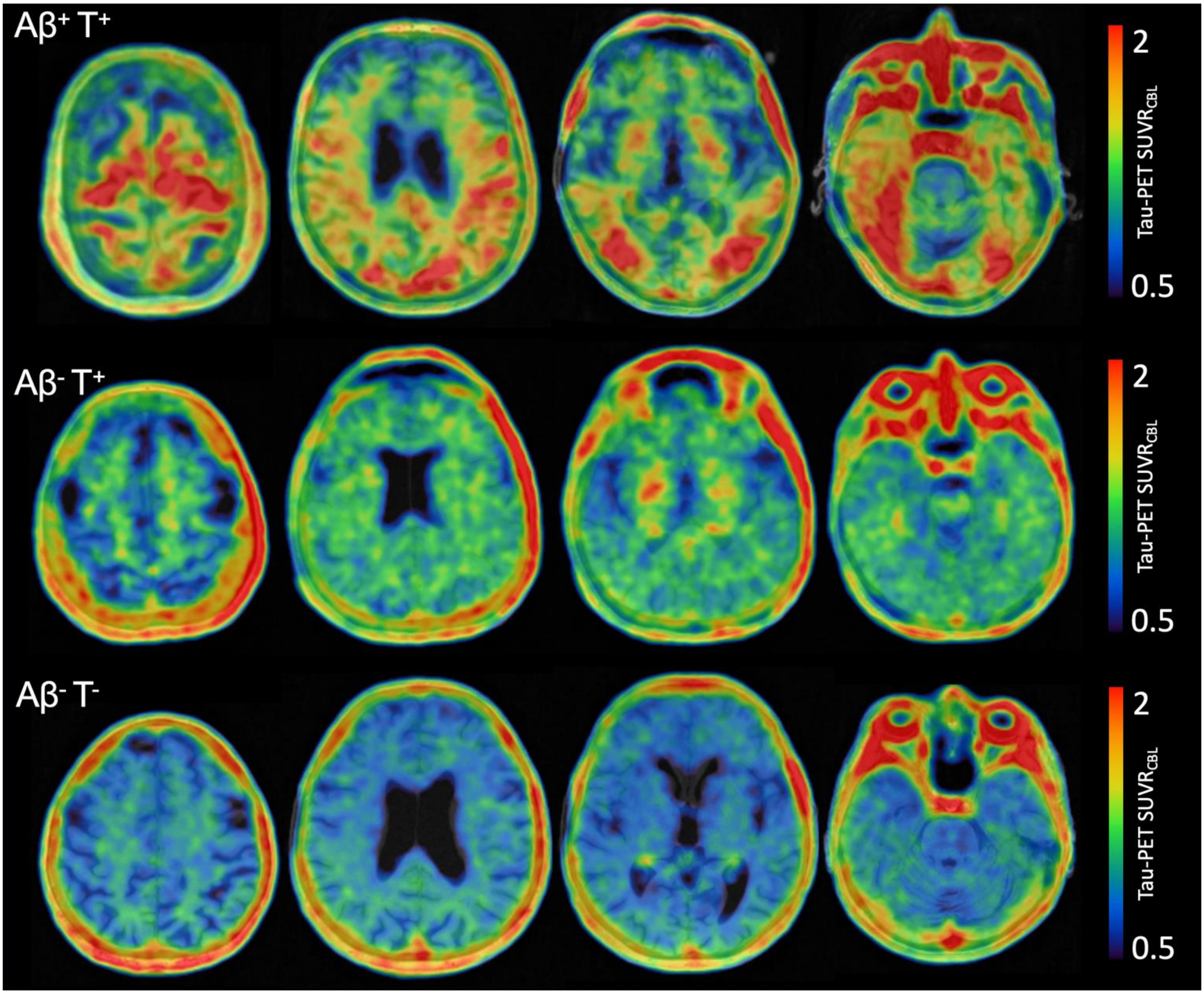

Supplementary Figure 2. Representative [^{18}F]PI-2620 tau-PET SUVR images across biomarker-defined CBS subgroups

SUVR: standardized uptake value ratio, CBL: cerebellar grey matter, Aβ: beta-amyloid, AD: Alzheimer's disease, PET: positron emission tomography.

Four axial sections are shown for each of three representative individuals (left-to-right within each panel: superior→inferior). Images are late-phase static SUVR maps (20–40 min) intensity-scaled to the inferior cerebellar grey matter reference (common scale 1.0–2.0), processed and visually read as described in the Methods (per the standardized algorithm for [^{18}F]PI-2620 visual assessment). The same color scale is applied across panels.

Aβ^{+}/Tau^{+} (AD-like): Widespread cortical tracer uptake with additional subcortical involvement, consistent with mixed 3R/4R tau distribution in amyloid-positive CBS.

Aβ^{-}/Tau^{+} (tau-predominant): Subcortical-predominant uptake with visually positive signal in the basal ganglia (notably globus pallidus/putamen) and absent Alzheimer-typical cortical pattern.

Aβ^{-}/Tau^{-} (Aβ- and tau-negative): Absence of specific tracer uptake in cortical and subcortical target regions.

.

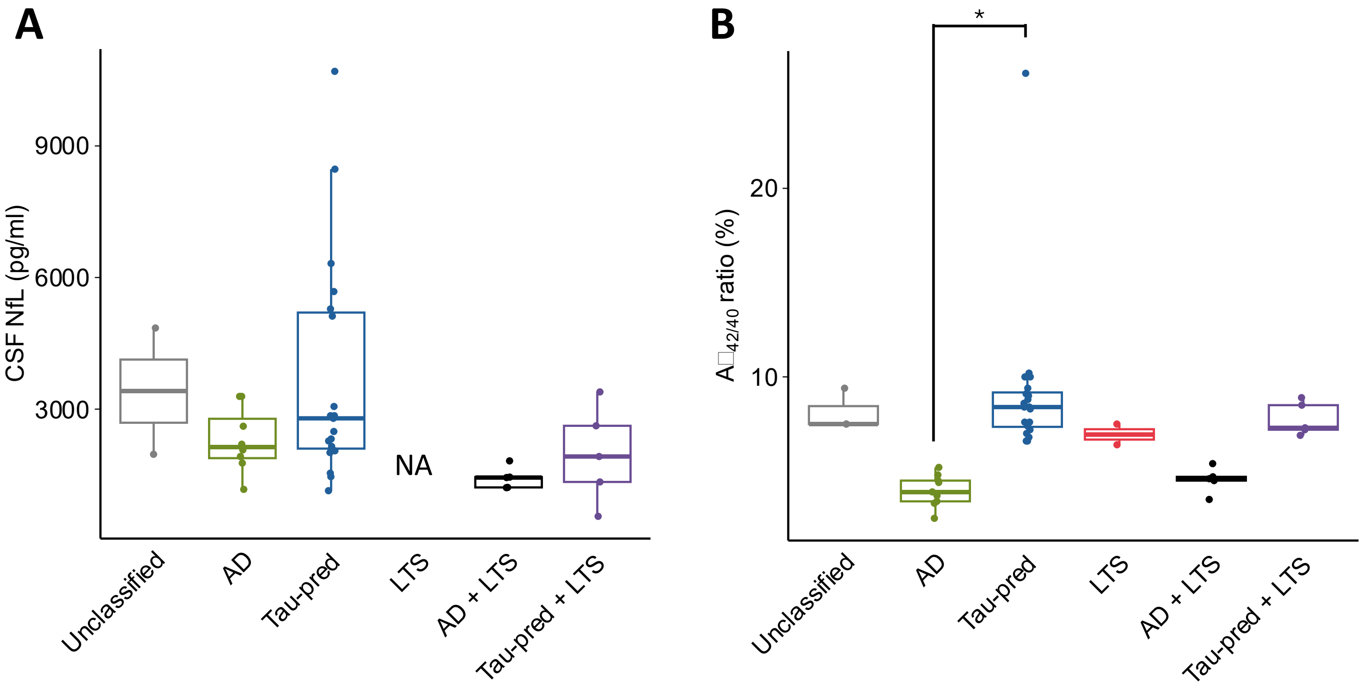

Supplementary Figure 3: Association between the biomarker-defined disease status and selected CSF biomarkers

AD: Alzheimer Disease, tau-pred: tau-predominant pathology, LTS: Lewy-Type Synucleinopathy, CSF: cerebrospinal fluid, NfL: Neurofilament light chain, NA: not applicable.

The boxplots display the distribution of NfL values (**A**) and A$\beta_{42/40}$ ratio values (**B**) across the six groups with different presumed underlying pathologies. Due to the relatively small group sizes, ANCOVA models corrected for age, sex and disease duration yielded mostly insignificant p-values, except for a significantly lower A$\beta_{42/40}$ ratio in AD compared to the tau-pred group ($p=0.01$, Tukey's post-hoc test).

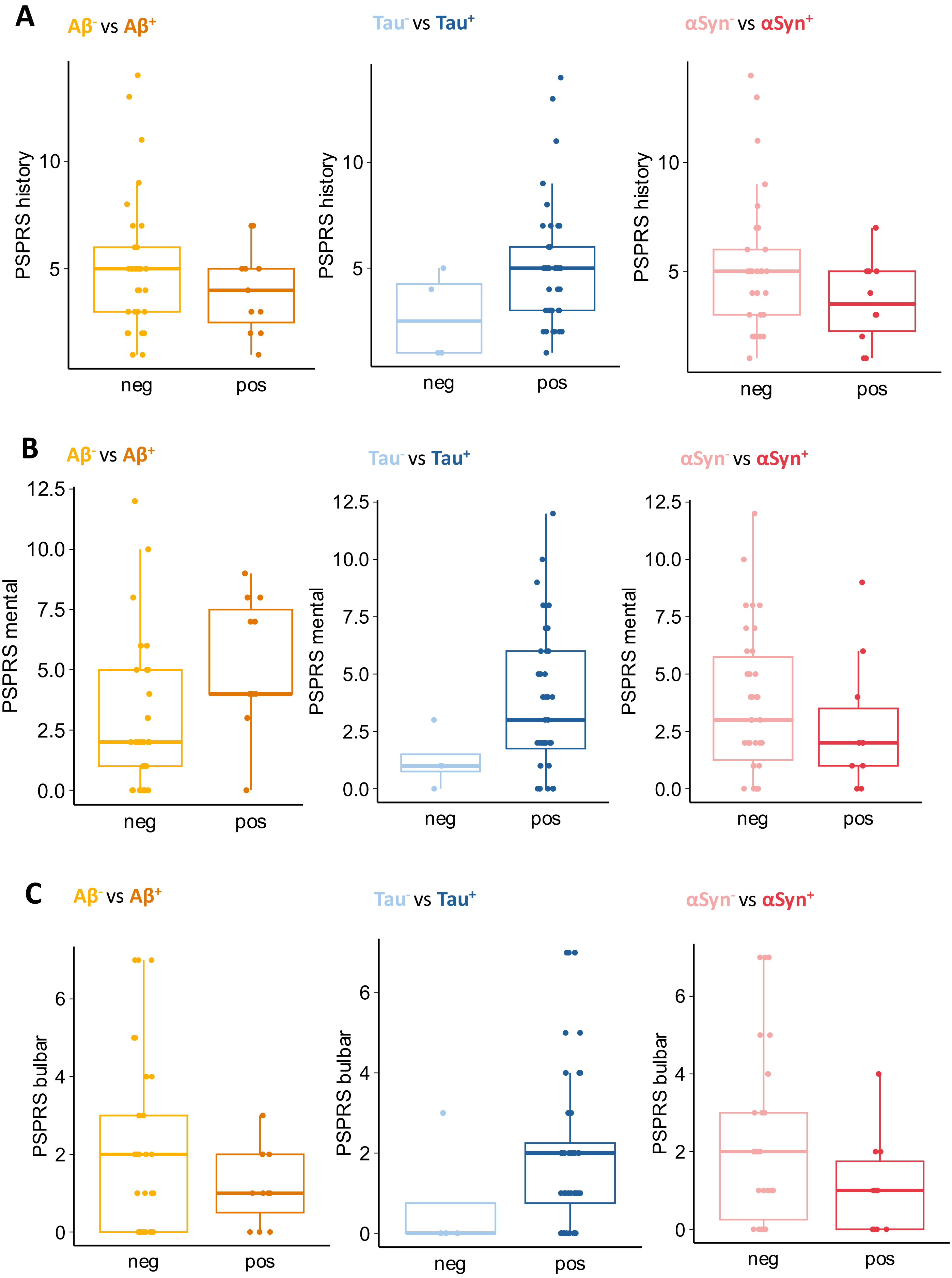
A
Aβ⁻ vs Aβ⁺
Tau⁻ vs Tau⁺
αSyn⁻ vs αSyn⁺
PSPRS history
10
5
neg
pos
B
Aβ⁻ vs Aβ⁺
Tau⁻ vs Tau⁺
αSyn⁻ vs αSyn⁺
PSPRS mental
12.5
10.0
7.5
5.0
2.5
0.0
neg
pos
C
Aβ⁻ vs Aβ⁺
Tau⁻ vs Tau⁺
αSyn⁻ vs αSyn⁺
PSPRS bulbar
6
4
2
0
neg
pos

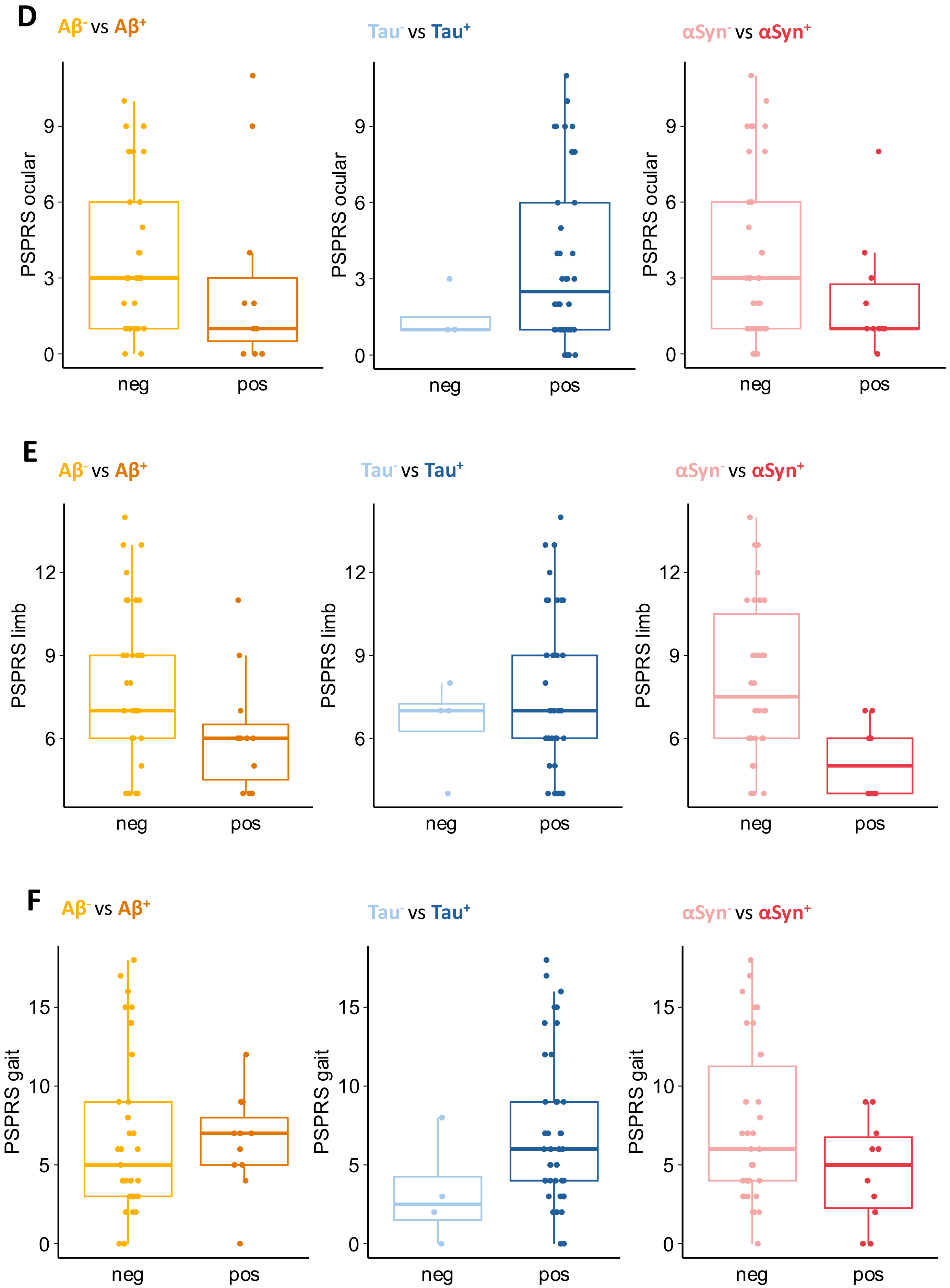

D
Aβ⁻ vs Aβ⁺
Tau⁻ vs Tau⁺
αSyn⁻ vs αSyn⁺
PSPRS ocular
0
3
6
9
neg
pos
E
Aβ⁻ vs Aβ⁺
Tau⁻ vs Tau⁺
αSyn⁻ vs αSyn⁺
PSPRS limb
6
9
12
neg
pos
F
Aβ⁻ vs Aβ⁺
Tau⁻ vs Tau⁺
αSyn⁻ vs αSyn⁺
PSPRS gait
0
5
10
15
neg
pos

Supplementary Figure 4: Association between the biomarker status (Aβ, Tau, αSyn) and PSPRS subscores

$A\beta^+$: Amyloid-β-positive, $A\beta^-$: Amyloid-β-negative, αSyn^+: α-Synuclein-positive, αSyn^-: α-Synuclein-negative, Tau^+: Tau-positive, Tau^-: Tau-negative, PSPRS: Progressive Supranuclear Palsy Rating Scale (higher scores indicate more severe impairment).

The boxplots visualize the distribution of PSPRS subscores across $A\beta^+$ and $A\beta^-$, or Tau^+ and Tau^- or αSyn^+ and αSyn^- groups. Because these sub-score comparisons lie outside the study's central, hypothesis-driven analyses, no inferential statistics are reported; the figure is provided solely to give a descriptive visual overview.

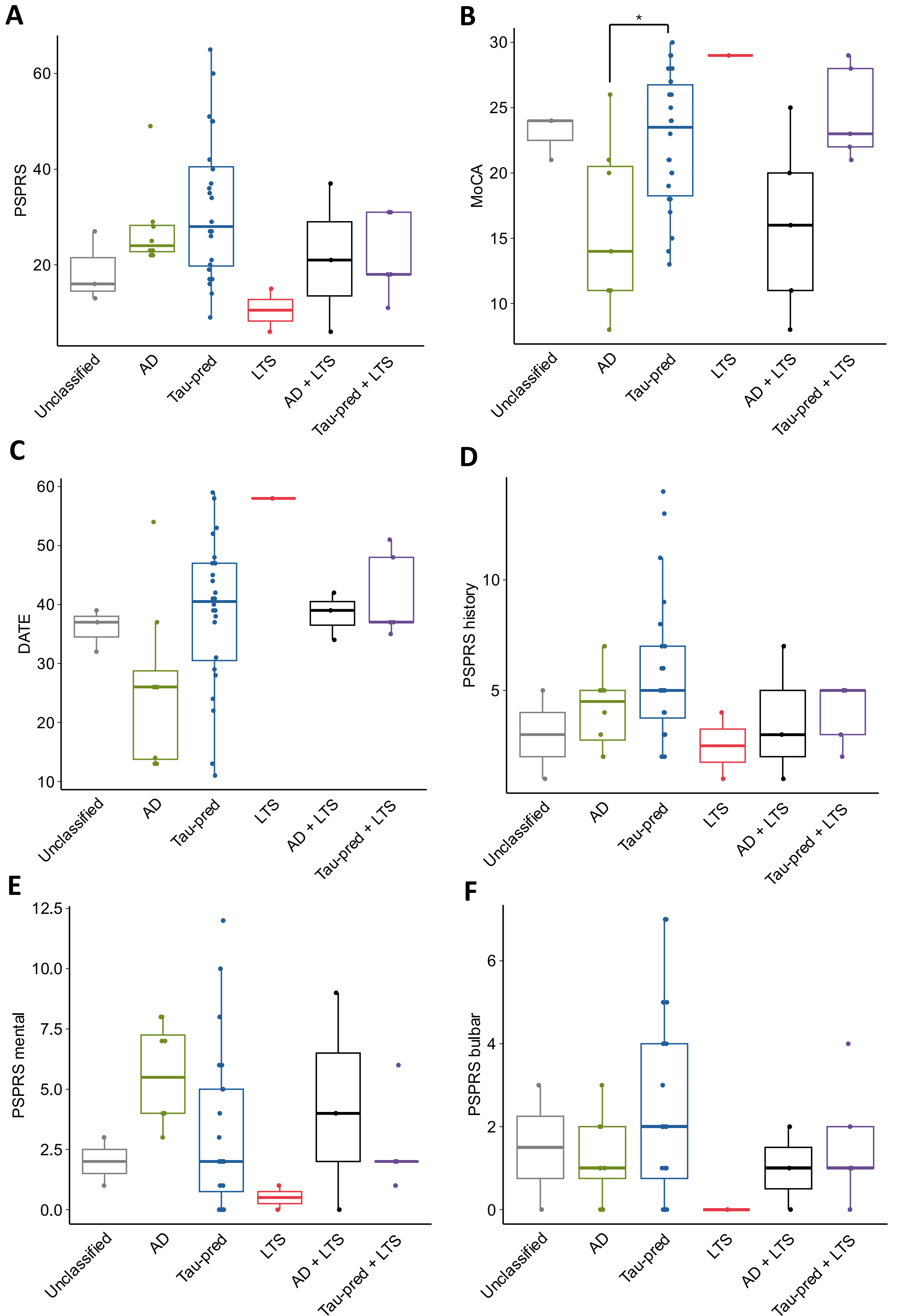
A
PSPRS
60
40
20
Unclassified
AD
Tau-pred
LTS
AD + LTS
Tau-pred + LTS
B
MoCA
*
30
25
20
15
10
C
DATE
60
50
40
30
20
10
D
PSPRS history
10
5
E
PSPRS mental
12.5
10.0
7.5
5.0
2.5
0.0
F
PSPRS bulbar
6
4
2
0

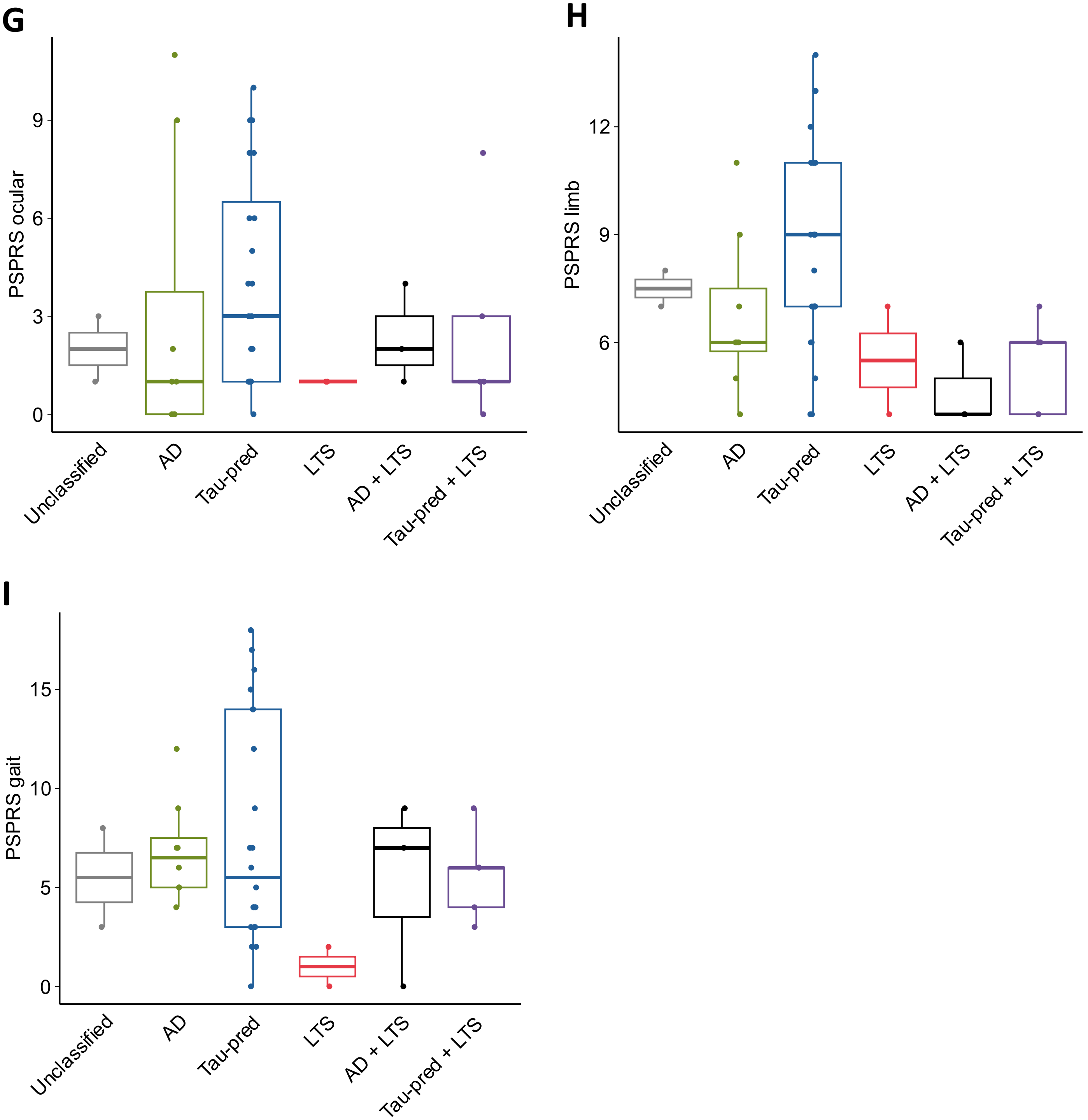

Supplementary Figure 5: Association between the biomarker-defined disease status and clinical scores (PSPRS, MoCA, DATE)

AD: Alzheimer Disease, tau-pred: tau-predominant pathology, LTS: Lewy-Type Synucleinopathy, PSPRS: Progressive Supranuclear Palsy Rating Scale (higher scores indicate more severe impairment), MoCA: Montreal Cognitive Assessment (higher scores indicate better cognitive performance), DATE: Dementia Apraxia Test (higher scores indicate better performance).

The boxplots visualize the distribution of PSPRS total scores (**A**), MoCA scores (**B**), DATE scores (**C**) and PSPRS subscores (**D-I**) across the six groups with different presumed underlying pathologies. Due to the relatively small group sizes, ANCOVA models corrected for age, sex and disease duration yielded mostly insignificant p-values, except for a significantly lower MoCA in AD compared to the tau-pred group (p=0.035, Tukey's post-hoc test).

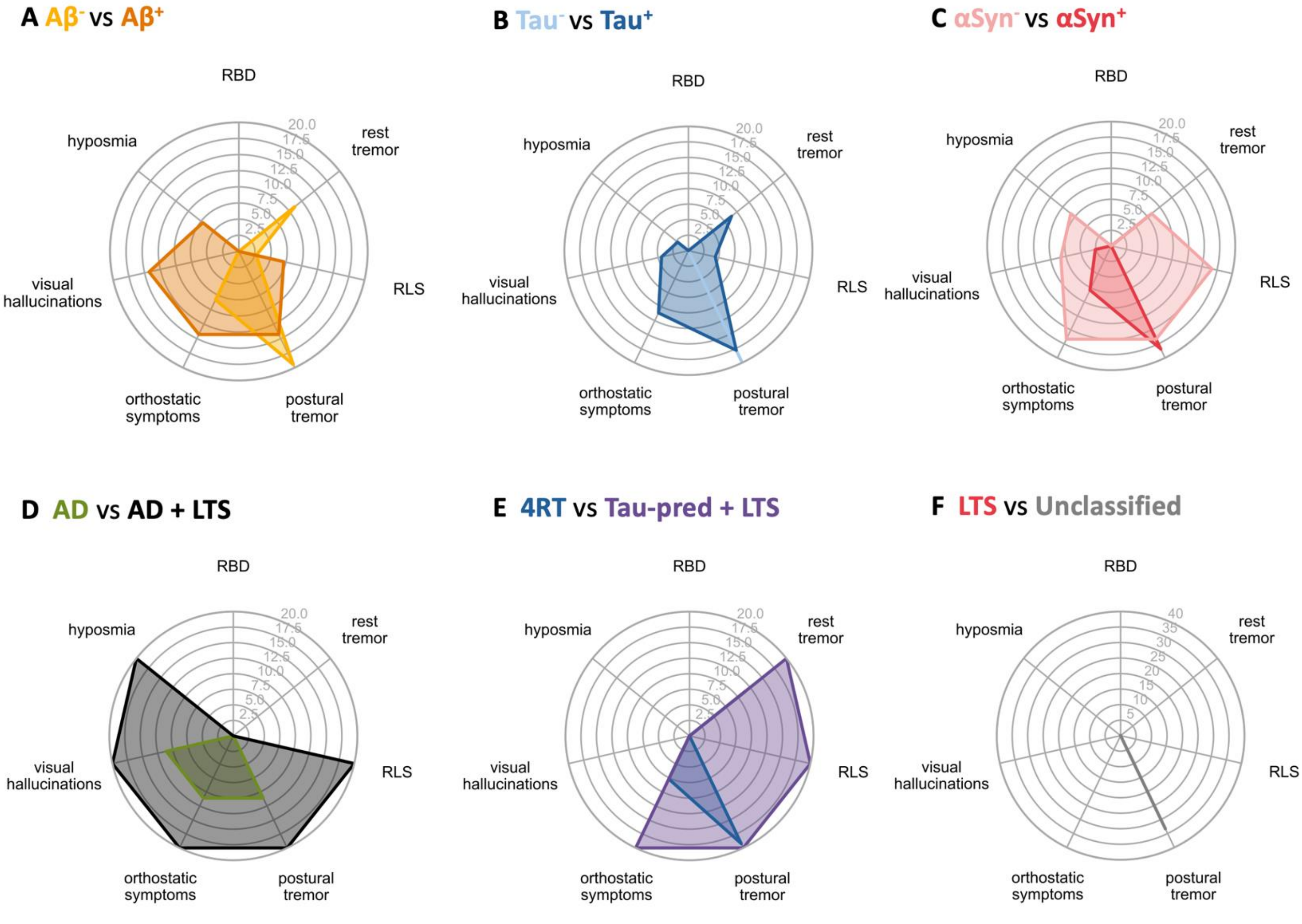

Supplementary Figure 6: Presence of clinical features in biomarker-based subgroups of CBS

Aβ+: Amyloid-β-positive, Aβ-: Amyloid-β-negative, αSyn+: α-Synuclein-positive, αSyn-: α-Synuclein-negative, Tau+: Tau-positive, Tau-: Tau-negative, AD: Alzheimer's Disease pathology, RLS: restless legs syndrome, RBD: rapid eye movement sleep behavior disorder, AD: Alzheimer Disease, tau-pred: tau-predominant pathology, LTS: Lewy-Type Synucleinopathy.

The radar plots illustrate the percentage of individuals exhibiting specific clinical features within each group. Each axis of the radar plot represents a clinical feature, and the distance from the center reflects the frequency in % of that feature within the respective group. The closer a point is to the edge, the more prevalent the feature is in that group. Panel (**B**): Of the 5 Tau- cases, one exhibited postural tremor (20%). No other displayed symptoms were noted. Panel (**F**): Among the 3 unclassified cases, one exhibited postural tremor (33.3%). No other displayed symptoms were noted in this group. Due to the relatively small group sizes, fisher exact tests yielded no significant results.

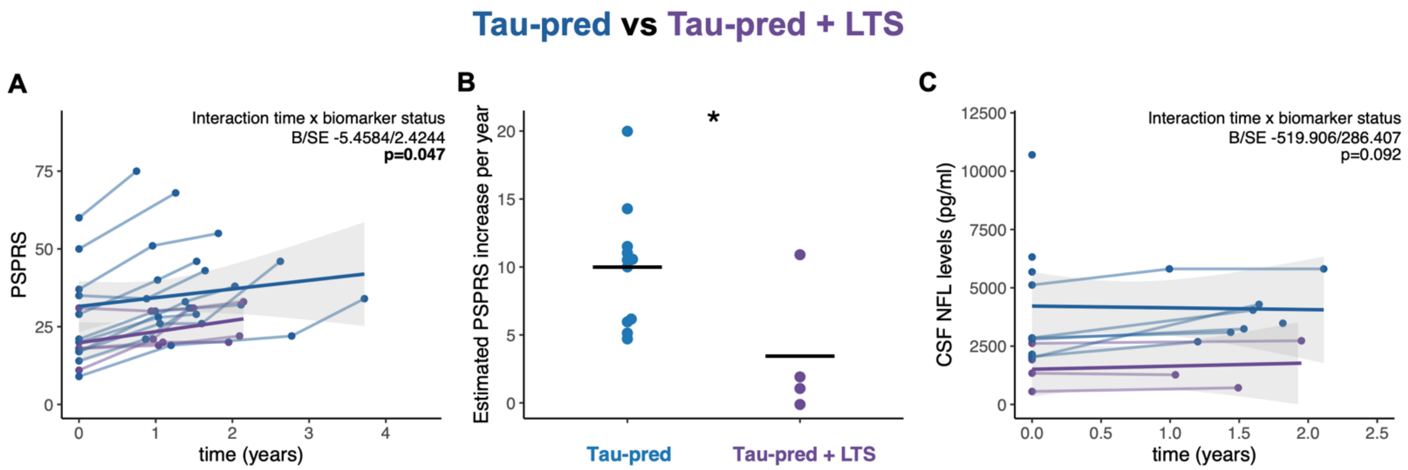

Supplementary Figure 7: Interaction of the biomarker-defined disease status and disease progression

αSyn+: α-Synuclein-positive, αSyn-: α-Synuclein-negative, Tau+: Tau-positive, Tau-: Tau-negative, tau-pred: tau-predominant pathology, LTS: Lewy-type Synucleinopathy, PSPRS: Progressive Supranuclear Palsy Rating Scale (higher scores indicate more severe impairment), NfL: Neurofilament light chain.

Line plots illustrating clinical trajectories on the PSPRS (**A**), and NfL (**C**) stratified by tau-pred and tau-pred+ LTS. Linear model fits (i.e., least squares line) are indicated together with 95% CIs. Statistics are based on linear mixed models controlling for age, sex, disease duration, number of follow-up visits per patient, and random slope and intercept. For visualization, regression fits were split into dichotomous biomarker status to illustrated disease trajectories relative to biomarker abnormality; however, interactions were computed using continuous measures. The estimated increase of PSPRS per year for each patient is shown in Panel **B**.
