# Supplementary material for: A Biomarker‐Based Classification of Corticobasal Syndrome
Source: Mov Disord. 2025 Oct 6;41(1):129–42. doi: 10.1002/mds.70070 (PMC12882055; doi:10.1002/mds.70070)
Supplement: Supplementary file 2 — Table S1. Logistic regression analysis of Aβ, Tau, and αSyn status on disease severity and biomarkers. [file MDS-41-129-s001.docx]

# Supplementary materials

## Supplementary methods

### Participants and clinical assessments

All study-related procedures have been approved by the LMU Munich ethics committee (ethics-applications: 23-0602, 17-569, and 19-022) and the German radiation protection authorities (BfS-application: Z5−22464/2017-047-K-G). All patients have provided written informed consent for all study related procedures including clinical assessment, lumbar puncture and PET imaging in accordance with the Declaration of Helsinki and its amendments. Patients did not receive compensation for study participation. All individuals who collected data as part of this project had received special training for all study-related procedures. Inclusion criteria were (1) an age above 45 years; (2) stable pharmacotherapy for at least 1 week before PET examination; (3) a negative family history for neurodegenerative diseases; and (4) no severe neurologic or psychiatric disorders other than CBS. Clinical diagnoses were re-evaluated at all follow-up visits.

## Neurofilament Light Chain in CSF

The NfL concentration in blood or CSF has been identified as a surrogate parameter for the activity of ongoing neurodegeneration in various neurological diseases^1^. NfL levels were quantified in CSF samples using a commercial SIMOA kit (#103345; Quanterix, Billerica, MA) using the Simoa HD-X analyzer. All samples underwent only 1 freeze/thaw cycle and were analyzed blinded to clinical information.

## Genetic testing

Participants were not systematically screened for mutations as part of the study protocol unless aged 60 or younger for the most common juvenile-onset and adult-onset genetic PD mutations (FBXO7, GBA1, LRRK2, PARK7, PINK1, PRKN, SNCA, VPS13C, VPS35, ATP13A2, DNAJC6, FBXO7, SLC6A3, SYNJ1).

## Data availability

The data are available upon reasonable request to the corresponding author from qualified academic investigators if data transfer is in agreement with EU legislation on general data protection regulation and decisions made by the Ethical Review Board of the LMU University Hospital.

## References

1. Khalil M, Teunissen CE, Lehmann S, et al. Neurofilaments as biomarkers in neurological disorders - towards clinical application. *Nat Rev Neurol*. 2024;20(5):269-287. doi:10.1038/s41582-024-00955-x

Supplementary Table

**Supplementary Table 1: Logistic Regression Analysis of Aβ, Tau, and αSyn status on disease severity and biomarkers**

| **Model: biomarker status ∼ independent variable + age + disease duration + sex** | | | | | | |
| --- | --- | --- | --- | --- | --- | --- |
| **Biomarker** | **logistic regression results** | **independent variables** | | | | |
|  |  | **NfL CSF** | **Aβ ratio** | **PSPRS** | **MoCA** | **DATE** |
| **Aβ status** | coefficient | -0.001 | -39.748 | -0.0037 | -0.279 | -0.084 |
|  | p-value | 0.069 | NA (perfect separation) | 0.907 | ****0.005** | ***0.027** |
|  | McFadden’s Pseudo-R-squared | 0.268 | 1.000 | 0.138 | 0.416 | 0.271 |
| **Tau status** | coefficient | 0.000043 | -0.0072 | 0.132 | -0.117 | -0.040 |
|  | p-value | 0.950 | 0.964 | 0.061 | 0.336 | 0.413 |
|  | McFadden’s Pseudo-R-squared | 0.556 | 0.085 | 0.294 | 0.185 | 0.196 |
| **αSyn status** | coefficient | -0.002 | -0.050 | -0.122 | 0.028 | 0.063 |
|  | p-value | ***0.050** | 0.757 | ***0.033** | 0.677 | 0.119 |
|  | McFadden’s Pseudo-R-squared | 0.467 | 0.165 | 0.269 | 0.169 | 0.177 |

This table presents the results of logistic regression models examining the association between biomarker status (Aβ, Tau, and αSyn status) and various clinical measures of disease severity (PSPRS, MoCA, DATE, and selected biomarkers including NfL CSF and Aβ ratio). The models control for age, disease duration, and sex. Coefficients represent the log-odds of biomarker status as a function of the independent variables. McFadden’s Pseudo-R-squared values indicate the goodness of fit for each model. Significant results are denoted by *p < 0.05 and **p < 0.01.

Abbrevations: Aβ^+^: Amyloid-β-positive, Aβ^-^: Amyloid-β-negative, αSyn^+^: αSynuclein-positive, αSyn^-^: αSynuclein-negative, Tau^+^: Tau-positive, Tau^-^: Tau-negative, PSPRS: Progressive Supranuclear Palsy Rating Scale, MoCA: Montreal Cognitive Assessment, DATE: Dementia Apraxia Test, NfL: Neurofilament Light Chain, NA: not applicable.

Supplementary Figures

### Supplementary Figure 1: Biomarker guided classification to corticobasal syndrome patients

Aβ+: Amyloid-β-positive, Aβ-: Amyloid-β-negative, αSyn+: α-Synuclein-positive, αSyn-: α-Synuclein-negative, Tau+: Tau-positive, Tau-: Tau-negative, tau-pred: tau-predominant pathology, AD: Alzheimer’s Disease pathology, LTS: Lewy-type Synucleinopathy.

This figure presents alternative, unbiased biomarker-guided approaches to classify corticobasal syndrome (CBS) patients into six distinct groups based on Aβ, Tau, and αSyn status. Panel A shows an approach starting with Tau stratification, followed by αSyn, and then Aβ. Panel B starts with αSyn stratification, followed by Aβ, and then Tau. In both cases, patients are categorized based on their biomarker status: Patients were screened for Aβ by Amyloid-PET or CSF and denoted as Aβ^-^ in light yellow or Aβ^+^ in dark yellow if one of these measurements showed pathological results. Tau-PET was employed to stratify patients into Tau^-^ in light blue and Tau^+^ in dark blue. αSyn seed amplification assay from CSF was employed to categorize patients into αSyn^-^ and αSyn^+^ displayed in light red and dark red, respectively. Despite varying the order of biomarker stratification, the resulting classification consistently identifies the same six groups, each representing a distinct presumed underlying pathology.

### Supplementary Figure 2. Representative [¹⁸F]PI-2620 tau-PET SUVR images across biomarker-defined CBS subgroups

SUVR: standardized uptake value ratio, CBL: cerebellar grey matter, Aβ: beta-amyloid, AD: Alzheimer’s disease, PET: positron emission tomography.

Four axial sections are shown for each of three representative individuals (left-to-right within each panel: superior→inferior). Images are late-phase static SUVR maps (20–40 min) intensity-scaled to the inferior cerebellar grey matter reference (common scale 1.0–2.0), processed and visually read as described in the Methods (per the standardized algorithm for [¹⁸F]PI-2620 visual assessment). The same color scale is applied across panels.

**Aβ^+^/Tau^+^ (AD-like):** Widespread cortical tracer uptake with additional subcortical involvement, consistent with mixed 3R/4R tau distribution in amyloid-positive CBS.

**Aβ^−^/Tau^+^ (tau-predominant):** Subcortical-predominant uptake with visually positive signal in the basal ganglia (notably globus pallidus/putamen) and absent Alzheimer-typical cortical pattern.

**Aβ^−^/Tau^−^ (Aβ- and tau-negative):** Absence of specific tracer uptake in cortical and subcortical target regions.

### Supplementary Figure 3: Association between the presumed biomarker-defined disease status and selected CSF biomarkers

AD: Alzheimer Disease, tau-pred: tau-predominant pathology, LTS: Lewy-Type Synucleinopathy, CSF: cerebrospinal fluid, NfL: Neurofilament light chain.

The boxplots display the distribution of NfL values (**A**) and Aβ_42/40_ ratio values (**B**) across the six groups with different presumed underlying pathologies. Due to the relatively small group sizes, ANCOVA models corrected for age, sex and disease duration yielded mostly insignificant p-values, except for a significantly lower Aβ_42/40_ ratio in AD compared to the tau-pred group (p=0.01, Tukey‘s post-hoc test).

### Supplementary Figure 4: Association between the biomarker status (Aβ, Tau, and αSyn) and PSPRS subscores

Aβ^+^: Amyloid-β-positive, Aβ^-^: Amyloid-β-negative, αSyn^+^: α-Synuclein-positive, αSyn^-^: α-Synuclein-negative, Tau^+^: Tau-positive, Tau^-^: Tau-negative, PSPRS: Progressive Supranuclear Palsy Rating Scale (higher scores indicate more severe impairment).

The boxplots visualize the distribution of PSPRS subscores across Aβ^+^ and Aβ^-^, or Tau^+^ and Tau^-^ or αSyn^+^ and αSyn^-^ groups. Because these sub-score comparisons lie outside the study’s central, hypothesis-driven analyses, no inferential statistics are reported; the figure is provided solely to give a descriptive visual overview.

### Supplementary Figure 5: Association biomarker-defined disease status and clinical scores (PSPRS, MoCA, DATE)

### AD: Alzheimer Disease, tau-pred: tau-predominant pathology, LTS: Lewy-Type Synucleinopathy, PSPRS: Progressive Supranuclear Palsy Rating Scale (higher scores indicate more severe impairment), MoCA: Montreal Cognitive Assessment (higher scores indicate better cognitive performance), DATE: Dementia Apraxia Test (higher scores indicate better performance).

### The boxplots visualize the distribution of PSPRS total scores (**A**), MoCA scores (**B**), DATE scores (**C**) and PSPRS subscores (**D-I**) across the six groups with different presumed underlying pathologies. Due to the relatively small group sizes, ANCOVA models corrected for age, sex and disease duration yielded mostly insignificant p-values, except for a significantly lower MoCA in AD compared to the tau-pred group (p=0.035, Tukey‘s post-hoc test).

Supplementary Figure 6: Presence of clinical features in biomarker-based subgroups of CBS

Aβ+: Amyloid-β-positive, Aβ-: Amyloid-β-negative, αSyn+: α-Synuclein-positive, αSyn-: α-Synuclein-negative, Tau+: Tau-positive, Tau-: Tau-negative, AD: Alzheimer‘s Disease pathology, RLS: restless legs syndrome, RBD: rapid eye movement sleep behavior disorder, AD: Alzheimer Disease, tau-pred: tau-predominant pathology, LTS: Lewy-Type Synucleinopathy.

The radar plots illustrate the percentage of individuals exhibiting specific clinical features within each group. Each axis of the radar plot represents a clinical feature, and the distance from the center reflects the frequency in % of that feature within the respective group. The closer a point is to the edge, the more prevalent the feature is in that group. Panel (**B**): Of the 5 Tau- cases, one exhibited postural tremor (20%). No other displayed symptoms were noted. Panel (**F**): Among the 3 unclassified cases, one exhibited postural tremor (33.3%). No other displayed symptoms were noted in this group. Due to the relatively small group sizes, fisher exact tests yielded no significant results.

### Supplementary Figure 7: Interaction of the biomarker-defined disease status and disease progression

### αSyn^+^: α-Synuclein-positive, αSyn^-^: α-Synuclein-negative, Tau^+^: Tau-positive, Tau^-^: Tau-negative, tau-pred: tau-predominant pathology, LTS: Lewy-type Synucleinopathy, PSPRS: Progressive Supranuclear Palsy Rating Scale (higher scores indicate more severe impairment), NfL: Neurofilament light chain.

Line plots illustrating clinical trajectories on the PSPRS (**A**), and NfL (**C**) stratified by tau-pred and tau-pred + LTS. Linear model fits (i.e., least squares line) are indicated together with 95% CIs. Statistics are based on linear mixed models controlling for age, sex, disease duration, number of follow-up visits per patient, and random slope and intercept. For visualization, regression fits were split into dichotomous biomarker status to illustrated disease trajectories relative to biomarker abnormality; however, interactions were computed using continuous measures. The estimated increase of PSPRS per year for each patient is shown in Panel **B**.
